# Sequencing of methylase-accessible regions in integral circular extrachromosomal DNA reveals differences in chromatin structure

**DOI:** 10.1186/s13072-021-00416-5

**Published:** 2021-08-23

**Authors:** Weitian Chen, Zhe Weng, Zhe Xie, Yeming Xie, Chen Zhang, Zhichao Chen, Fengying Ruan, Juan Wang, Yuxin Sun, Yitong Fang, Mei Guo, Yiqin Tong, Yaning Li, Chong Tang

**Affiliations:** 1 College of Life Sciences, University of Chinese Academy of Sciences, Beijing, 100049 China; 2 BGI Genomics, BGI-Shenzhen, Shenzhen, 518083 China; 3 Department of Biology, Cell Biology and Physiology, University of Copenhagen 13, 2100 Copenhagen, Denmark; 4 Nantong University, Nantong, 226000 China; 5 Nephrosis Precision Medicine Innovation Center, University of Beihua School of Medicine, Jilin City, 132011 China

**Keywords:** ecDNA, Chromatin accessibility, Methylation, m^6^A, Methyltransferase

## Abstract

**Background:**

Although extrachromosomal DNA (ecDNA) has been intensively studied for several decades, the mechanisms underlying its tumorigenic effects have been revealed only recently. In most conventional sequencing studies, the high-throughput short-read sequencing largely ignores the epigenetic status of most ecDNA regions except for the junctional areas.

**Methods:**

Here, we developed a method of sequencing enzyme-accessible chromatin in circular DNA (CCDA-seq) based on the use of methylase to label open chromatin without fragmentation and exonuclease to enrich ecDNA sequencing depth, followed by long-read nanopore sequencing.

**Results:**

Using CCDA-seq, we observed significantly different patterns in nucleosome/regulator binding to ecDNA at a single-molecule resolution.

**Conclusions:**

These results deepen the understanding of ecDNA regulatory mechanisms.

**Supplementary Information:**

The online version contains supplementary material available at 10.1186/s13072-021-00416-5.

## Introduction

Finding a cure for cancer has been a challenge for various reasons, such as oncogene amplification, tumor evolution, and genetic heterogeneity [9, 31, 36]. Recently, it has been demonstrated that circular extrachromosomal DNA (ecDNA) plays a critical role in carcinogenesis, as it promotes oncogene amplification [40], drives tumor evolution, and contributes to genetic heterogeneity [28, 37, 38]. Circular ecDNA is arranged next to chromatin in a circular structure, featuring the head-to-tail junctional sequence and distal homologous genome sequence. The cancer-specific ecDNA may have an average size of 1.3 MB [4]. Although ecDNA has been known since 1964 [28], the elucidation of its role has been slow due to the lack of adequate molecular analytical techniques [2].

The development of new techniques, including computational advances, enabled genetic and epigenetic studies of ecDNA, and attempts have been made to identify ecDNA from sequencing data using improved algorithms from specificity and sensitivity. Most algorithms, such as Circle-Map [30], AmpliconArchitect [7], and CIRC_finder [21], relied on the detection of the ecDNA junction sequence and enabled ecDNA identification in numerous cancer tissues [18, 21, 29, 37], aging cells [15], blood plasma [20, 41], and healthy somatic tissues [26]. However, due to the rareness of ecDNA in sequencing data, these approaches require the enrichment of ecDNA molecules; for example, circular DNA is obtained by the digestion of linear DNA with nucleases, followed by rolling circle amplification [25]. To further improve the accuracy of ecDNA detection, the long-read sequencing technology has been used to verify the ecDNA junction structure [6, 24]. However, functional epigenetic studies of ecDNA are currently lacking. Given the increasing awareness of ecDNA and its role in oncogene expression, understanding genome-wide ecDNA chromatin state and transcription status is essential. The most recent and advanced theory proposed by Wu et al. offers insights into highly accessible chromatin region and high expression of oncogenes located within these regions in ecDNA using the assay for transposase-accessible chromatin sequencing (ATAC-seq), and chromatin immunoprecipitation sequencing (Chip-seq) [40]. Moreover, few studies have examined genome-wide ecDNA epigenome, because it is difficult to analyze ecDNA junction structure and epigenome information simultaneously.

Moreover, ATAC-seq and Chip-seq analyses are based on the peak calling algorithm of populated fragments, and they do not consider the molecular heterogeneity. Therefore, there are benefits for expanding sequencing length and achieving single molecular resolution of the ecDNA epigenome owing to the limitations due to the short-read sequencing and short fragmentation required for ATAC-seq [3] and MNase-seq [32]. Our study was prompted by the existing long-read sequencing methods for assessing chromatin state, such as MeSMLR-seq [39], SAMOSA [1], nanoNOMe-seq [22], SMAC-seq [33], and fiber-seq [35]. We used the N6-methyladenosine (m^6^A) methyltransferase EcoGII to soft label accessible chromatin regions without fragmentation and named this method “sequencing of enzyme-accessible chromatin in circular DNA” (CCDA-seq). Using this method, we enriched ecDNA by digesting the linear genome using nuclease. Nanopore sequencing accurately detected the m^6^A-probed ecDNA regions of accessible chromatin and junctional structure properties simultaneously in the long range. Using CCDA-seq, we found a high diversity of ecDNA regions of accessible chromatin and their coordination with distal regulators at a single-molecule resolution, which has not been reported before.

## Results

### CCDA-seq comprehensively maps accessible chromatin in ecDNA at a multikilobase scale

**Fig. 1:**
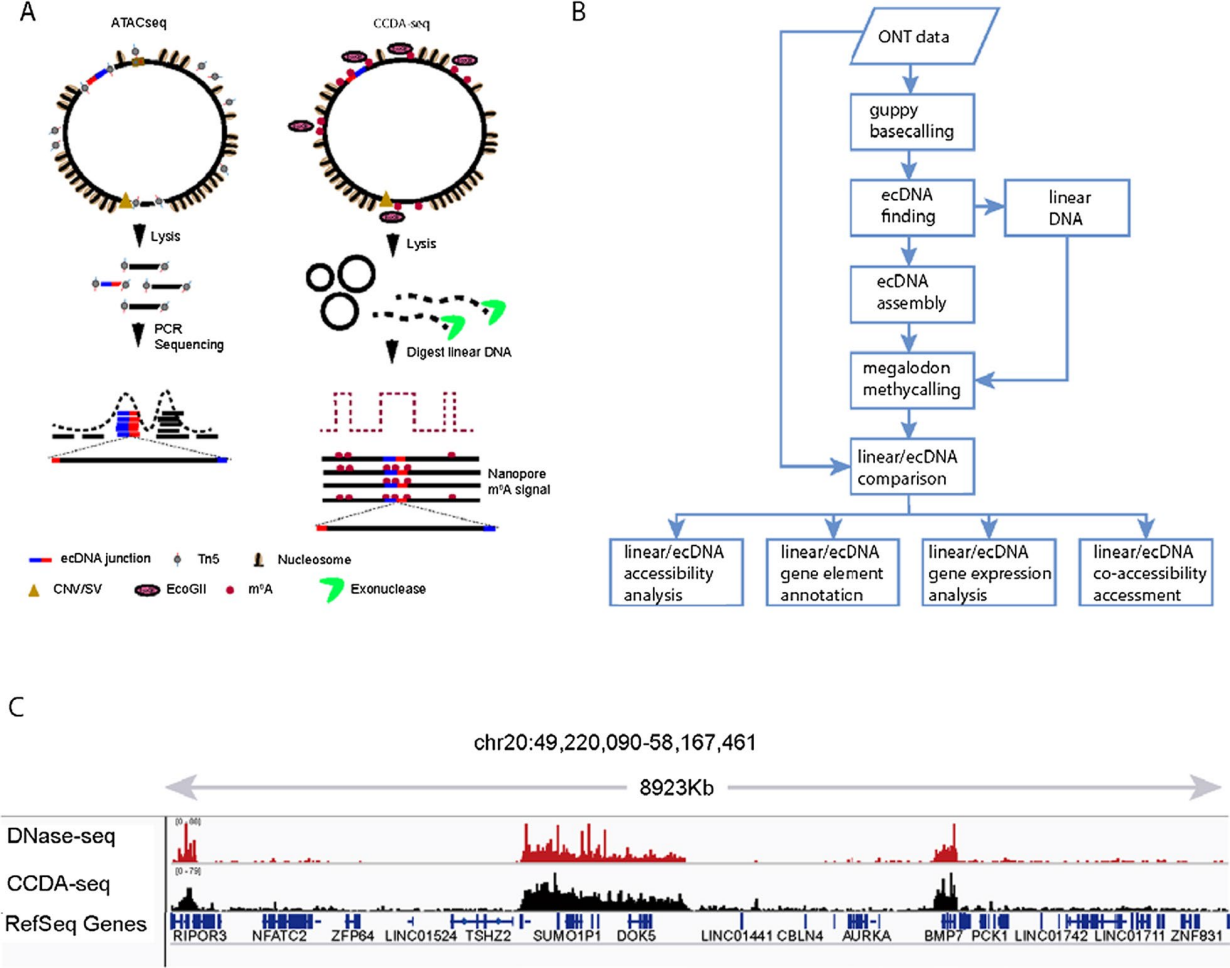
CCDA-seq for profiling chromatin accessibility and nucleosome position in ecDNAs. **A** Intact chromatin was treated with m^6^A methyltransferase (EcoGII), which preferentially methylates DNA bases in the open chromatin region on ecDNAs and linear DNAs. High molecular weight DNA was then isolated and subjected to exonuclease digestion to remove partially linear DNAs. The remaining DNA molecules were subjected to nanopore library construction and nanopore sequencing. The data were aligned to the hg19 genome to identify ecDNAs based on head-to-tail pattern. The methylated bases were used to reconstruct nucleosomes in ecDNAs and other linear DNAs. In contrast, the ATAC-seq used the transposon to attack the open chromatin. The tagmentated short fragments were amplified and subjected to NGS. The short reads were aligned with genome to identify ecDNA bases. The mapped reads were calling as peaks representing the open chromatin region. **B** CCDA-seq bioinformatics pipeline is illustrated. The signal data were processed through guppy base calling to generate sequence. The sequences were aligned to the genome to identify linear DNAs and ecDNAs. We assembled the ecDNA sequence reference. Based on the ecDNA and linear DNA references, we used Megalodon to call the m^6^A sites based on ecDNA and linear DNA sequences. Then, we performed the accessibility analysis, gene element annotation, gene expression analysis, and co-accessibility assessment. **C** Large aggregate CCDA-seq signal enrichments match closely with DNase-seq accessibility peaks. (Chr20: 49220090–58167461)

ecDNA plays an important role in tumorigenesis due to the high accessibility of its chromatin and carried oncogenes [40]. Conventional approaches to study chromatin accessibility are based on the concept that chromatin protects the bound sequence from attack by transposase (Fig. 1A) or MNase [32]. In ATAC-seq, the open, accessible genome region is first preferentially tagged using transposase, followed by next-generation sequencing (NGS) (Fig. 1A). However, this method is not employed in most integral ecDNA chromatin studies due to the homologous ecDNA/genome sequences, making the distinction between ecDNA and linear genome DNA difficult. In general, previous studies of ecDNA chromatin based on NGS of short reads only observed the chromatin status in the junction region (200 bp around the junction) and bioinformatically analyzed other distal ecDNA areas because of limitations of the techniques used (> 200 bp to junction regions) (Fig. 1A). To solve these problems, we built a generalized framework based on the concept of the MeSMLR-seq (Wang et al. 2019), SAMOSA (Abdulhay et al. 2020), SMAC-seq [33], and fiber-seq [35]. We applied soft labeling with the m^6^A methyltransferase EcoGII that preferentially methylates the adenosine in the openly accessible DNA region without fragmentation by a transposase (Fig. 1A). To improve the ecDNA capturing efficiency, the exonuclease was introduced to remove the linear genome DNA [8]. The integral ecDNA was sequenced by nanopore sequencing, and the probed m^6^A was detected [33]. By analysing the generated data, we first identified ecDNA molecules by head-to-tail junction locations and by dynamically mapping the segments of sequences to the genome (Fig. 1B). Based on the head-to-tail junction locations, we then reassembled the partial ecDNA sequences as the new reference and identified the m^6^A signal based on the reassembled ecDNA sequence to prevent signal bias in the junction region (Fig. 1B).

The statistical analysis showed that the read length was between 10 and 100 kb, which was 50× broader than the junctional region observed in conventional ATAC-seq (Additional file 1: Figure S1). The long-read feature also makes the nanopore sequencing method optimal for applications, such as structure variation (SV), copy number variation (CNV), and ecDNA identification with better sensitivity and specificity [14]. As expected, 80% of ecDNA molecules detected in our CCDA-seq could be validated through PCR (Additional file 1: Figure S2). ecDNA and residual linear DNA accounted, respectively, for 0.9% and 99.1% of the total sequencing reads (Additional file 1: Figure S3) after exonuclease treatment, in which ecDNA was enriched ninefold (Additional file 1: Figure S4). To further explore ecDNA functionality, we assembled ecDNAs to reconstruct the full length of ecDNA, the length of which ranged from 2 kb to 1 MB (Additional file 1: Figure S5). Seventy-five percent of ecDNA contained genes (Additional file 1: Figures S6, S7), suggesting the translational function of ecDNA.

The m^6^A probability distribution in Megalodon showed two distinct peaks for the treated sample. The distribution of the narrow peak with lower m^6^A probability (mean = 0.49) was similar to the background noise distribution (Additional file 1: Figure S8). Therefore, we set m^6^A methylation probability over 0.53 as the cutoff for the true m^6^A signal (Additional file 1: Figure S8). The real positive cutoff value was set at 0.53, and the m^6^A calling specificity and sensitivity were 0.99 and 0.92, respectively (Additional file 1: Figure S4). The residual linear DNA was used as internal control for validation using published ATAC-seq data [11]. CCDA-seq achieved consistency and coherence with ATAC-seq data in various resolutions (Fig. 1C, Additional file 1: Figures S9, S10). The m^6^A labeling deviation was inversely proportional to the m^6^A ratio and strongly reduced to 0.015 in m^6^A-enriched region (Additional file 1: Figure S11). The impact of the exonuclease treatment and reproducibility have been also investigated (Additional file 1: Figure S12). These characteristics of CCDA-seq were critical for effectively measuring the accessibility of chromatin in linear and circular DNA molecules in the multikilobase range.

Another remarkable feature of CCDA-seq is that it enables investigation of the ecDNA chromatin status at a single-molecule resolution, at which the single base m^6^A probability varied from 0.6 to 1. (Additional file 1: Figure S13). In practice, the resolution of accessible chromatin regions was around 200 bp. We adopted a Bayesian procedure to aggregate methylation probabilities and derived the accurate single-molecule accessibility calls over windows of arbitrary size (Additional file 1: Figure S13). In summary, CCDA-seq offers attractive features in terms of the elucidation of the integral ecDNA chromatin status in the multikilobase range at a single-molecule resolution.

### Diverse patterns of ecDNA chromatin accessibility

Evidence from other studies that utilized ATAC-seq and Chip-seq suggests that the active chromatin status and highly accessible ecDNA chromatin may be associated with high levels of oncogene transcription [40]. To distinguish ecDNA molecules from linear DNA molecules in ATAC-seq and Chip-seq, it is necessary to screen out the short reads (~ 200 bp) spanning the non-homologous end-joining ecDNA sequence. One problem with these approaches is the potential bias to neglect the distal regions due to focusing on the ~ 200 bp reads neighboring ecDNA junctional sequences. CCDA-seq, as a long-read technology, may facilitate precise ecDNA detection [6, 14, 26] and observation of the distal chromatin status in integral ecDNA. We obtained an extensive catalog of 12,997 different ecDNA molecules formed from chromosomal breakpoints between 0.05 and 100 kb (Additional file 2: Table S1). Gene ontology (GO) analysis of the genes harbored by these ecDNA molecules revealed significant enrichment in the GO terms GTPase-related activity, channel activity, and nucleoside-triphosphatase activity, i.e., processes playing essential roles in cancer progression (Additional file 1: Figure S14) [13, 16]. RNA-seq data analysis showed that there were 340 highly expressed ecDNA genes (25% rank), 464 moderately expressed genes (25–75% rank), and 589 genes with low expression (75–100% rank), indicating that not all ecDNA genes are highly expressed.

**Fig. 2:**
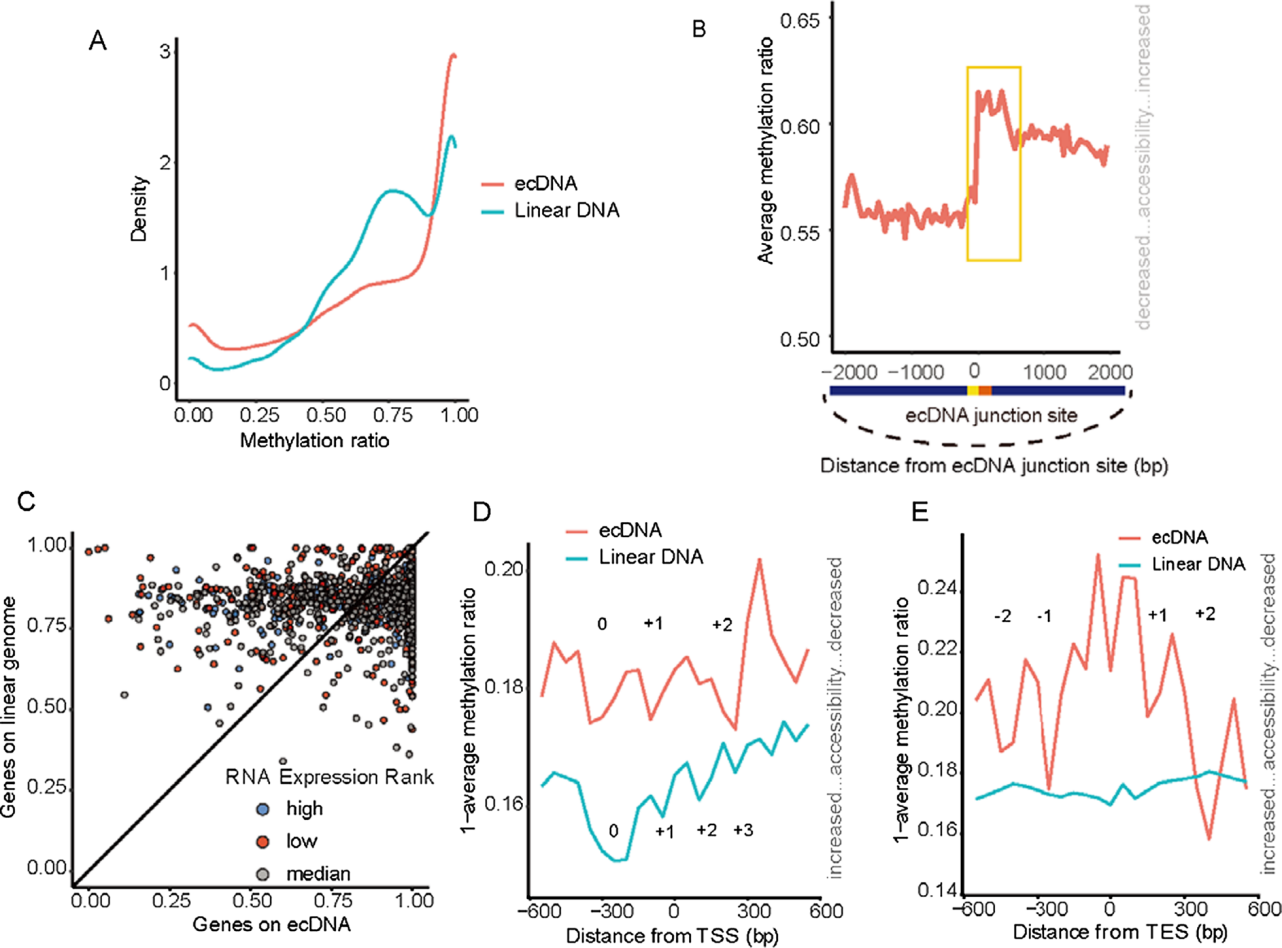
ecDNA and linear DNA have the different chromatin accessibility pattern. **A** Density distribution of the methylation ratio in ecDNA and linear DNA. **B** Average chromatin status around ecDNA neighboring regions is illustrated. The junction site and its right neighboring regions demonstrate the more open chromatin. **C** Average methylations of gene regions on ecDNA and linear DNA (from TSS to TES). The genes were classified into two groups: (I) the genes on linear DNA have more open chromatin structure than ecDNA carried genes (above centerline); (II) the ecDNA carried genes have more open chromatin structure than the genes on linear DNA (below centerline). **D** Average CCDA-seq profile around transcription start site on ecDNAs and linear DNA. **E** Average CCDA-seq profile around the transcription end site in the ecDNA and linear DNAs (aggregated over 50-bp windows sliding every 5 bp; the sequencing depth was normalized for ecDNA and linear DNA; see Method for details)

By comparing the average chromatin accessibility between ecDNA and homologous linear DNA, we found that the ecDNA chromatin is twofold more accessible than that the linear DNA chromatin (Fig. 2A). These findings reinforce the general notion that ecDNA amplification results in higher oncogene transcription [40], coupled with the enhanced chromatin accessibility in the junctional region. The CCDA-seq data were subjected to the detailed mapping of the ecDNA chromatin status. We found that chromatin in the ecDNA junctional areas is significantly more accessible than in other junction distal regions (Fig. 2B). This is an interesting finding, as it suggests that the conclusions drawn by observing only the junctional areas after the conventional ATAC-seq may be biased and not necessarily relate to the whole ecDNA chromatin. We calculated average fractions of m^6^A methylation from the gene transcription start site (TSS) to the gene transcription end site (TES) on each gene-spanning read. A pairwise scatter plot of the average accessibility between ecDNA genes and linear genome genes showed that 62% of gene regions are more accessible in the ecDNA than in the linear DNA (Fig. 2C) (considering that standard deviation is 5%). A comparison of ecDNA and linear DNA chromatin profiles around the TSS/TES (+/− 500 bp) revealed a significant difference in nucleosome depletion/occupancy patterns (Fig. 2D, E). The nucleosome organization may impact access to ecDNA (Fig. 2D, E). Considering that 62% of gene regions were more accessible in the ecDNA than in the linear DNA, we further plotted the chromatin structure around TSS/TES (+/− 500 bp) of these genes (Additional file 1: Figure S15). The formation of nucleosome depletion regions (NDRs) on linear DNA is restricted to 200 bp before TSSs. In contrast, the NDRs in ecDNA are distributed uniformly (Additional file 1: Figure S15). The other 36% of gene regions are more accessible in the linear DNA than in the ecDNA. The TSSs/TESs (+/− 500 bp) were also significantly more accessible in the linear DNA than in the ecDNA with different NDR patterns (Additional file 1: Figure S16). The formation of large NDRs was restricted to TSSs in the linear DNA, which was not observed in the ecDNA.

Another illustration of the complex interplay between chromatin states in the ecDNA and linear DNA relates to the transcriptional activity. The chromatin of the linear DNA active genes (top 25% rank) is largely devoid of nucleosomes on TSSs due to the extremely high transcription activity (Additional file 1: Figure S17). In contrast, the chromatin structure of the ecDNA active genes adopts a distinct conformation, implying that ecDNA is regulated by different mechanisms (Additional file 1: Figure S17). For the transcriptionally inactive genes, the stationary nucleosome states are shown in the linear DNAs (Additional file 1: Figure S18). In contrast, ecDNA molecules still have the active nucleosome organization in the regions of 300 bp before TSSs, suggesting that chromatin accessibility is necessary but not sufficient for the enhancer or promoter activity in the ecDNA (Additional file 1: Figure S18). In conclusion, ecDNA and linear DNA have significantly different nucleosome depletion/occupancy patterns in various conditions, suggesting their distinct gene regulatory mechanisms.

### Chromatin status in the ecDNA and linear genome DNA at a single-molecule resolution

The conventional ATAC-seq is based on statically calling the peak of the enriched read in a specific region [3]. Recent single-molecule and single-cell accessibility measurements suggested that ATAC-seq of cell populations represents an ensemble average of distinct molecular states [17]. An essential attribute of the CCDA-seq is a possibility to determine ecDNA chromatin accessibility at a single-molecule resolution by taking the advantage of small variance (Additional file 1: Figure S11) and increased cumulative probability in segments (Additional file 1: Figure S13). Measuring chromatin accessibility of the single linear DNA has also been done using SMAC-seq [33] and fiber-seq [35].

**Fig. 3:**
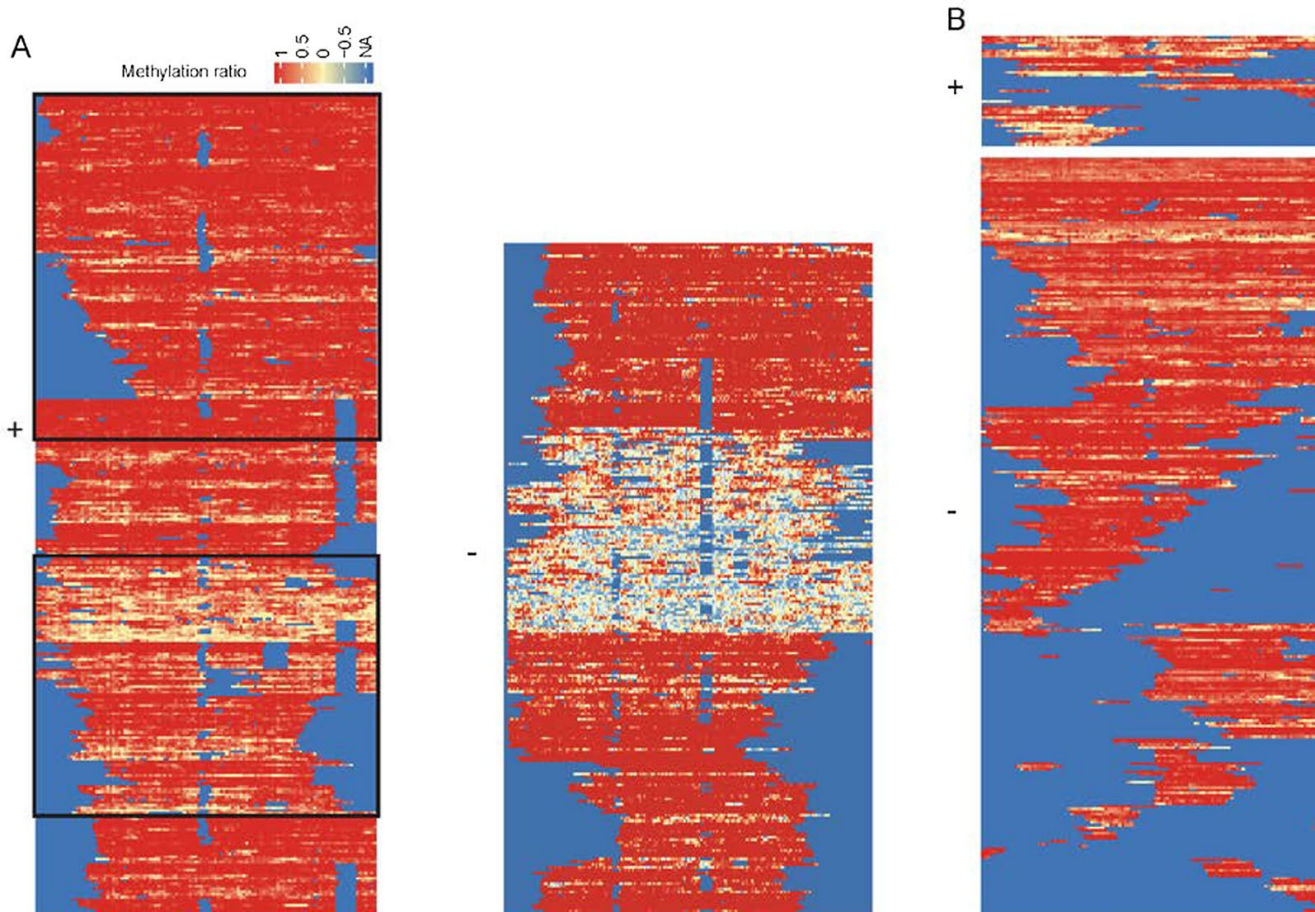
CCDA-seq reveals the distribution of alternative chromatin states in ecDNA arrays. **A** Shown are all reads covering the linear DNA region chr10: 42383201–42389201. The box highlights the active and inactive chromatins. **B** Shown are all reads covering the ecDNA region chr10: 42383201–42389201. The upper panel indicates the positive strand, and the lower panel indicates the negative strand

We then asked whether CCDA-seq could reveal multiple chromatin accessibility states in ecDNA. The chromatin structure of the linear DNA (chr10: 42383201–42389251) adopts two distinct conformations: an inactive nucleosomal state and a state largely devoid of nucleosomes due to extremely high transcription activity [5] (Fig. 3A). It is thought that ecDNA in the majority of cancer cells exhibits active nucleosome status. As expected, 70% of ecDNA molecules come from the very active chromatin state (Fig. 3A). We observed highly heterogeneous nucleosome depletion/occupancy patterns in ecDNA, and most chromatin molecules were not very active in the positive strand, suggesting distinct transcriptional regulation of ecDNA (Fig. 3B, upper panel). Some regulator enzymes may occupy the positive strand and restrict chromatin accessibility. The highly active ecDNA chromatin was also observed in other regions (Additional file 1: Figure S19). To avoid a conclusion biased by the heterogeneous activity of the methylase, other upstream and downstream regions were chosen as quality controls.

To further explore CCDA-seq resolution limits, we studied methylation patterns in more detail. In particular, we quantified strand-specific DNA accessibility and observed a strand-asymmetric DNA accessibility pattern in the linear genome (Additional file 1: Figure S19). The strand-asymmetric DNA accessibility pattern was also observed in ecDNA, and both strands displayed high heterogeneity (Fig. 3B, Additional file 1: Figure S19). This strand-specific heterogeneity in methylation potential within the nucleosome may inform about how transcription factors interact with nucleosome-associated DNA in vivo.

**Fig. 4:**
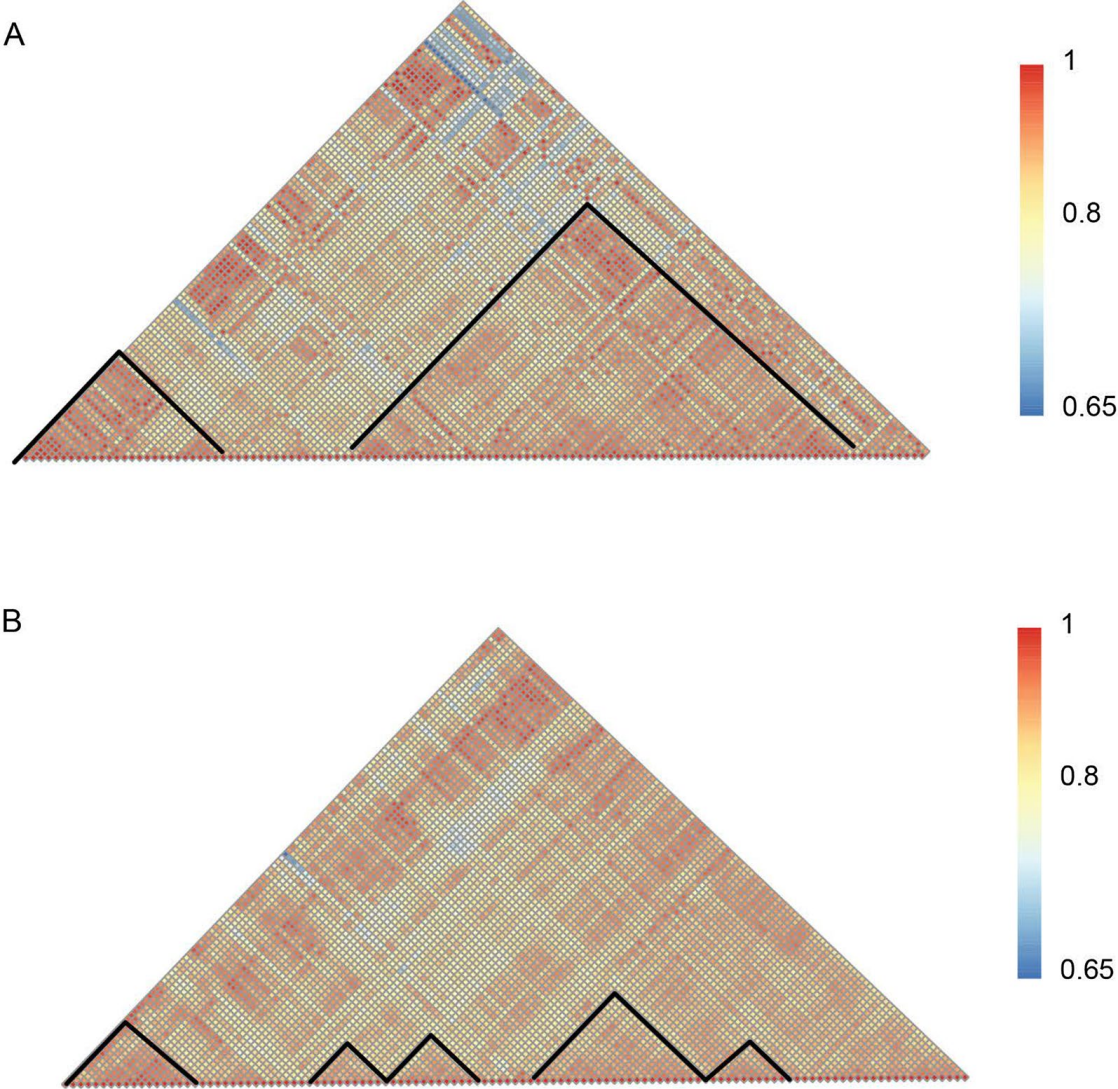
Chromatin co-accessibility profiles for the chr10: 42383201–42389201 show high and low correlation with ecDNA and linear DNA. **A** Chromatin co-accessibility profiles for the chr10: 42383201–42389201 show correlation and low correlation with ecDNA. Red indicates the positive correlation and blue indicates the low correlation. **B** Chromatin co-accessibility profiles for the chr10: 42383201–42389201 show correlation and low correlation on linear DNA

Wu et al. showed that ecDNA enables ultra-long range chromatin contact, permitting distant interactions with regulatory elements [40]. We next examined co-accessibility patterns in the ecDNA and linear genome DNA by assessing nucleosome positioning correlations. The nucleosomes have higher correlation values in the ecDNA than in the linear DNA (Fig. 4A, B,Additional file 1: Figure S20). Moreover, ecDNA and linear DNA adopt significantly different chromatin co-accessibility patterns (Fig. 4A, B; Additional file 1: Figure S20). Average co-accessibility profiles in the linear DNA revealed a detectable correlation between nucleosome positions up to two to three nucleosomes away. For the ecDNA, this correlation was further and up to 20 nucleosomes away (Fig. 4A, B; Additional file 1: Figure S20). These results agree with the high-resolution chromosome conformation capture (HiC) result [40] in that the ecDNA is characterized by the distant chromatin interaction. It was interesting to note that ecDNA demonstrated some ultra-distant anticorrelated states. Overall, ecDNA molecules were highly heterogeneous and exhibited remote chromatin interactions, suggesting their different regulation mechanisms compared to those acting on linear DNAs.

## Discussion

Understanding of ecDNA functions may prove to be essential for the elucidation of tumorigenesis mechanisms [28, 37, 38]. Many ecDNA molecules have been identified in various cancer tissues [18, 21, 29, 37]. There has been an increasing research focus on the status of ecDNA chromatin to resolve the problem of ecDNA oncogene amplification [40]. However, most studies focused on short sequencing reads with junctional sequences detected to avoid the false-positive identification of ecDNA and to precisely determine the ecDNA epigenetic status. A large subgroup (60%) of ecDNAs covered regions that are not unique in the reference genome, which complicated their identification [27]. In this study, we used nanopore sequencing to evaluate integral ecDNA chromatin accessibility in ecDNA long strands by applying m^6^A methyltransferase to label open chromatin without fragmentation. Consistent with the previously reported findings [40], most ecDNA genomic regions are more accessible than the linear genomic regions (Fig. 2A). For genetic regions, 63% of ecDNA molecules carried genes with more accessible chromatin structure than that of the linear DNA. However, in the remaining fraction of ecDNA (37%), chromatin of the gene regions was less accessible than in the corresponding linear DNA parts. Notably, the nucleosome depletion/occupancy patterns were significantly different between ecDNA and linear DNA. Our single-molecule resolution method allows footprinting of protein and nucleosome binding as well as determination of the epigenetic signature of chromatin accessibility. It is hoped that this study will contribute to more comprehensive understanding of the ecDNA epigenome regulation.

In our experiments, we treated DNA samples with an exonuclease that removed the majority of the linear DNA molecules and increased the sequencing depth for the ecDNA (0.9%). Some identified linear DNA molecules may be generated from the ecDNA homologous regions without junctions, but the likelihood of that was around 0.9%, which is negligible. Compared with the parameters in the non-digest direct sequencing, we only obtained 0.1% of ecDNA-related reads (Additional file 1: Figure S4). The circular ecDNA enrichment was ninefold. The exonuclease treatment not only improved ecDNA sequencing coverage, but also ecDNA detection specificity (Additional file 1: Figure S2). However, DNA purification process could damage large-size ecDNA molecules over 1 Mb [34]. Such damaged ecDNA could be digested during exonuclease digestion and missed in the sequencing. A method that gently purifies large DNA molecules would be preferable in further large-scale ecDNA studies.

Megalodon is the latest software (compared with Tombo) that was chosen for m^6^A signal calling. In the ecDNA m^6^A calling, Tombo ignored half of the sequences or lost most ecDNA molecules for unknown reasons (Additional file 1: Figure S21). The sensitivity of Tombo for ecDNA m6A signals was 83% less than that of Megalodon. Although Megalodon improved the sensitivity of ecDNA m^6^A calling, it did not address the issue of the false-positive m^6^A signal, that most adenosine bases could be recognized as m^6^A with a probability of 0.4–1 using Megalodon. The only known way to solve the false positive issue is to employ data training with negative control samples (Additional file 1: Figure S8). We used 0.53 as m^6^A probability cutoff, successfully discriminating the m^6^A and false-positive signals with sensitivity of 0.92 and specificity of 0.99. In general, Megalodon performed better in ecDNA analysis, and its specificity improved following data training.

In the sequencing data, we found that the methylated treated DNA generated more data than the non-methylated DNAs, which was not consistent with the SMAC-seq and fiber-seq data [33, 35]. The highly open chromatin with highly methylated sites may have been enriched using our method. In our laboratory experiments, we found that the heavily modified DNA was more resistant to exonuclease digestion and, therefore, enriched as a result of enzymatic treatment. The non-treated sample showed a lower overall methylation level (Additional file 1: Figure S22). However, the nucleosome occupancy positions were not significantly affected by the exonuclease treatment (Additional file 1: Figure S23). Moreover, in the strand-specific view, the reverse strand reads are generally less abundant than the positive strands. This may also be due to the different patterns of methylation of the positive and negative strands, which could result in different digestion efficiencies. This problem is usually overcome by increasing sequencing depth and using normalization methods. We also suggest that sequencing both treated and non-treated samples for ecDNA sequencing coverage will further improve quantification accuracy.

Only 63% of gene regions were within highly accessible chromatin in our experiments. However, Wu et al. showed using ATACseq technology that ecDNA molecules are mostly located in highly accessible chromatin [40]. When comparing results from all other regions with the published data, a good agreement was found in that 80% of ecDNA areas were highly accessible (Additional file 1: Figure S24). Most of the areas of highly accessible chromatin were distributed in the intron and intergenic regions (Additional file 1: Figure S25). The reasons for this remain unclear, but our results indicate that ecDNA has a highly open chromatin structure, especially in the intergenic and intronic regions.

## Conclusion

CCDA-seq is useful for studying the chromatin status of integral ecDNA, offering deep insights into the distinct mechanisms of ecDNA regulation. However, the ecDNA enrichment step requires exonuclease treatment, causing the loss of mega ecDNAs. It is assumed that future advances will help address the problems of DNA damage during the purification and the insufficient sequencing depth. The CCDA-seq will help the scientific community to understand different mechanisms of ecDNA regulation, especially in cancer development.

## Method

### Cell culture

Human mammary gland carcinoma cell line MCF-7 was obtained from ATCC. MCF-7 were grown in DMEM (Gibco 11995065) supplemented with 10% FBS (Gibco, 10099141), 0.01 mg/ml insulin(MedChemExpress, HY-P1156), and 1% penicillin–streptomycin (Gibco, 15140122). The cell line was regularly checked for mycoplasma infection (Yeasen, 40612ES25).

### Nuclei isolation and MTase treatment

Cells were grown to 70–80% confluency, and were collected by TrypLE (Gibco, 12604013). After 300×*g* centrifuge for 5 min, nuclei were isolated with lysis buffer (100 mM Tris–HCl, pH 7.4, 10 mM NaCl, 3 mM MgCl_2_, 0.1 mM EDTA, 0.5% CA630) for 5 min on ice. Nuclei were centrifuged at 300×*g* in wash buffer (100 mM Tris–HCl, pH 7.4, 10 mM NaCl, 3 mM MgCl_2_, 0.1 mM EDTA) at 4 degree, and washed twice for 5 min and counted.

1 × 10^6 intact nuclei were subjected to an m^6^A methylation reaction mixture containing 1 × Cutsmart buffer (NEB), 200U of non-specific adenine methyltransferase M.EcoGII (NEB, M0603S), 300 mM sucrose, and 96 μM S-adenosylmethionine (NEB, B9003S) in 500 μl volume. The reaction mixture was set up at a 37-degree thermomixer with shaking at 1000 rpm for 30 min. S-adenosylmethionine was replenished at 640 μM every 7.5 min at 7.5, 15, and 22.5 min into the reaction mixture. The reaction was stopped by adding an equal volume of stop buffer (20 mM Tris–HCl pH 7.4, 600 mM NaCl, 1% SDS, 10 mM EDTA). No methylation controls were treated in the same conditions without adding M.EcoGII in the reaction mixture. The samples were then treated with 20 μl of Proteinase K (20 mg/ml) at 55 degrees overnight, and the DNA was extracted with phenol: chloroform extraction and ethanol precipitation.

### ecDNA isolation, purification, and sequencing

ecDNA was isolated by Circle-Seq [25] method, which digested linear DNA with modifications. Briefly, 10 μg of M.EcoGII treated DNA was subjected to a reaction mixture containing 1 × plasmid-safe reaction buffer, 20U plasmid-safe ATP-dependent DNase (Lucigen, E3101K), 1 mM ATP, and nuclease-free water was supplemented to a final volume of 100 μl. The reaction mixture was incubated at 37 degrees for 7 days. Every 24 h, the reaction mixture was replenished by adding 20U plasmid-safe ATP-dependent DNase, 1 mM ATP, and 0.4 μl 10X plasmid-safe reaction buffer. Digested ecDNA was purified with 1.8X AMpure XP beads (Beckman Coulter).

Purified ecDNA was prepared for nanopore sequencing by ligation kit LSK-SQK108(ONT). The samples were 10 kb by Covaris G tubes, end-repaired and dA-tailed using NEBnext Ultra II end-repair module (NEB), followed by clean-up using 1.8X AMpure XP beads. Sequencing adaptors and motor proteins were ligated to end-repaired DNA fragments using blunt/TA ligase master mix (NEB), followed by clean-up using 0.4 × AMpure XP beads. 1ug adaptor-ligated samples per flow cell were loaded onto PRO-002 flowcells and run on PromethION sequencers for up to 72 h. Data were collected by MinKNOW v.1.14.

### Base-calling and linear DNA methylation calling

Reads from the ONT data were processed using Megalodon V2.2.9, which used Guppy base caller to base-calling, and Guppy model config res_dna_r941_min_modbases-all-context_v001.cfg released into the Rerio repository was used to identify DNA m^6^A methylation. Megalodon_extras was used to get per read modified_bases from the Megalodon basecalls and mappings results. To further explore the accurate threshold of methylation probability, a control sample with almost no m^6^A methylation was used as background noise, and the Gaussian mixture model was used to fit the methylation probability distribution generated by Megalodon.

### ecDNA calling

ONT Reads meet the following conditions were defined as ecDNA molecules performed by the inner mappy/minimap2 aligner [23]. (1) One segment (> 1 kb) of an ONT read was mapped to the genome at one site, and another segment (> 1 kb) was mapped to the genome at another site. (2) Two segments were mapped to the same chromosome. (3) Two segments were mapped to the same strand of the genome. (4) Two segments in a pair showed outward orientation.

### Nanopore ecDNA methylation calling

Due to ecDNA special structure, the m^6^A calling cannot be successfully performed by aligning to the reference genome, especially for junctional regions. The custom python script was used to assemble ecDNA reference genome sequences according to the table generated from the previous step. Considering that the read length might be longer than the ecDNA reference, the ecDNA reference was subsequently preprocessed by adding 10 M N to the ends to increase the mapping efficiency. The downstream step is performed in a similar way as linear DNA methylation calling.

### Circular DNAs assembly

Circular DNAs assembly was performed by Flye (v2.8.3-b1695) [19] assembler on the raw FASTQ files with the following settings: –genome-size 1G –meta–nano-raw [12]. Circular DNAs sequences were aligned to the hg19 genome by NCBI blastn(v2.2.28+).And we calculate the distribution and proportion of genes on Circular DNAs by custom script. Finally, the R packages circlize (v0.4.12.1004) [10] was used to visualize Circular DNAs.

### Annotation and methylation configuration

TES, TTS, CDS, and other gene elements were downloaded from UCSC Table Browser. In addition, the gene elements were processed into 50 bp bin for downstream analysis. Linear DNA and ecDNA were also processed to the size of 50 bp bin and sliding for 5 bp. The accessibility score over multi base-pair windows was calculated as methylation ratio = m^6^A bases in all covered reads under bin/ adenosine bases in all covered reads under the bin.

### RNA-seq data analysis

The RNA-seq data of MCF-7 was downloaded from the Gene Expression Omnibus (GEO) repository database with the accession number GSE71862. The gene expression was divided into three categories: high, medium, and low, representing 25%, 25–75%, and 75% gene expression rank, respectively.

### Co-accessibility assessment

To evaluate co-accessibility patterns along the genome, we applied COA as follows. Each chromosome in the genome was split into windows of size w. For each such window (*i*, *i* + *w*), we identified another window (*j*, *j* + *w*) such that the span (*i*, *j*, *w*) was covered by ≥ *N* reads. For each single spanning molecule *k*, accessibility scores (*A*) in each bin were then aggregated and binarized as described above. The local co-accessibility matrix between two windows was calculated:

$${\text{COA}}_{i,j,w} = {\text{ mean}}_{{\text{N}}} \left( {1 - \frac{{\left| {Ai,w { - } Aj,w} \right|}}{Ai,w + Aj,w}} \right)$$

## Supplementary Information

**Additional file 1.** Additional Figures.

**Additional file 2: Table S1.** The profiles of the ecDNAs.

## Data Availability

Nanopore raw data are available at China National GeneBank (CNGB) with project number of CNP0001720.

## Abbreviations

CCDA-seq: The sequencing of enzyme-accessible chromatin in circular DNA
ecDNA: Extrachromosomal DNA
m^6^A: N6-methyladenosine
ATAC-seq: The assay for transposase-accessible chromatin sequencing
Chip-seq: Chromatin Immunoprecipitation Sequencing
nanoNOMe-seq: Nanopore single-molecule Nucleosome Occupancy and Methylome sequencing
SMAC-seq: Single-molecule long-read accessible chromatin mapping sequencing assay
Mnase: Micrococcal nuclease
NGS: Next-generation sequencing
SV: Structure variation
CNV: Copy number variation
GO: Gene ontology
TSS: Transcription start site
TES: Gene transcription end site
NDRs: Nucleosome depletion regions
HiC: High-resolution chromosome conformation capture
ATACsee: Assay of transposase-accessible chromatin with visualization
CNGB: China National GeneBank
