# Supplementary material for: Sequencing of methylase-accessible regions in integral circular extrachromosomal DNA reveals differences in chromatin structure
Source: Epigenetics Chromatin. 2021 Aug 23;14:40. doi: 10.1186/s13072-021-00416-5 (PMC8383416; doi:10.1186/s13072-021-00416-5)
Supplement: Supplementary file 1 — Additional file 1. Additional Figures. [file 13072_2021_416_MOESM1_ESM.docx]

**
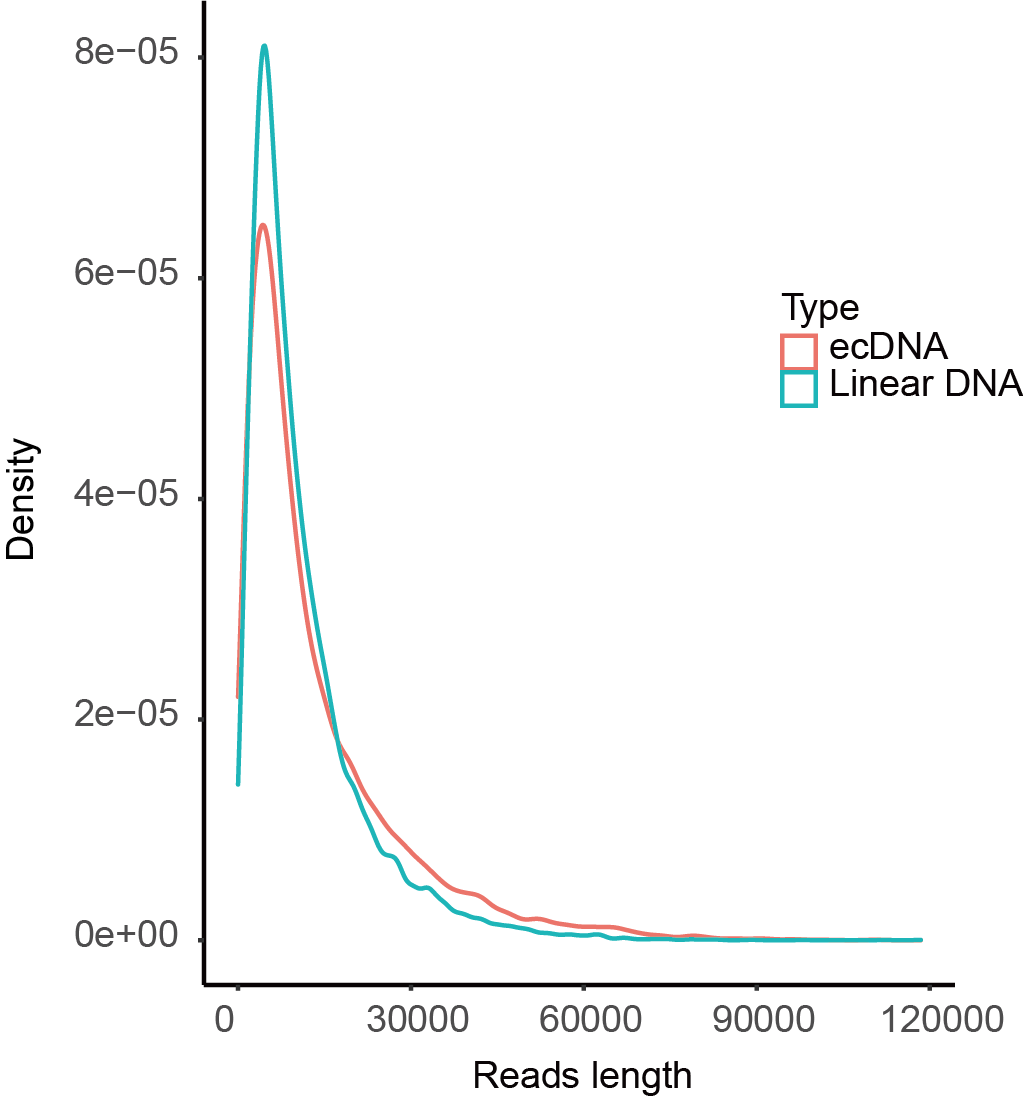
**

**Figure S1. Distribution of the lengths of reads identified as ecDNA or linear DNA.**


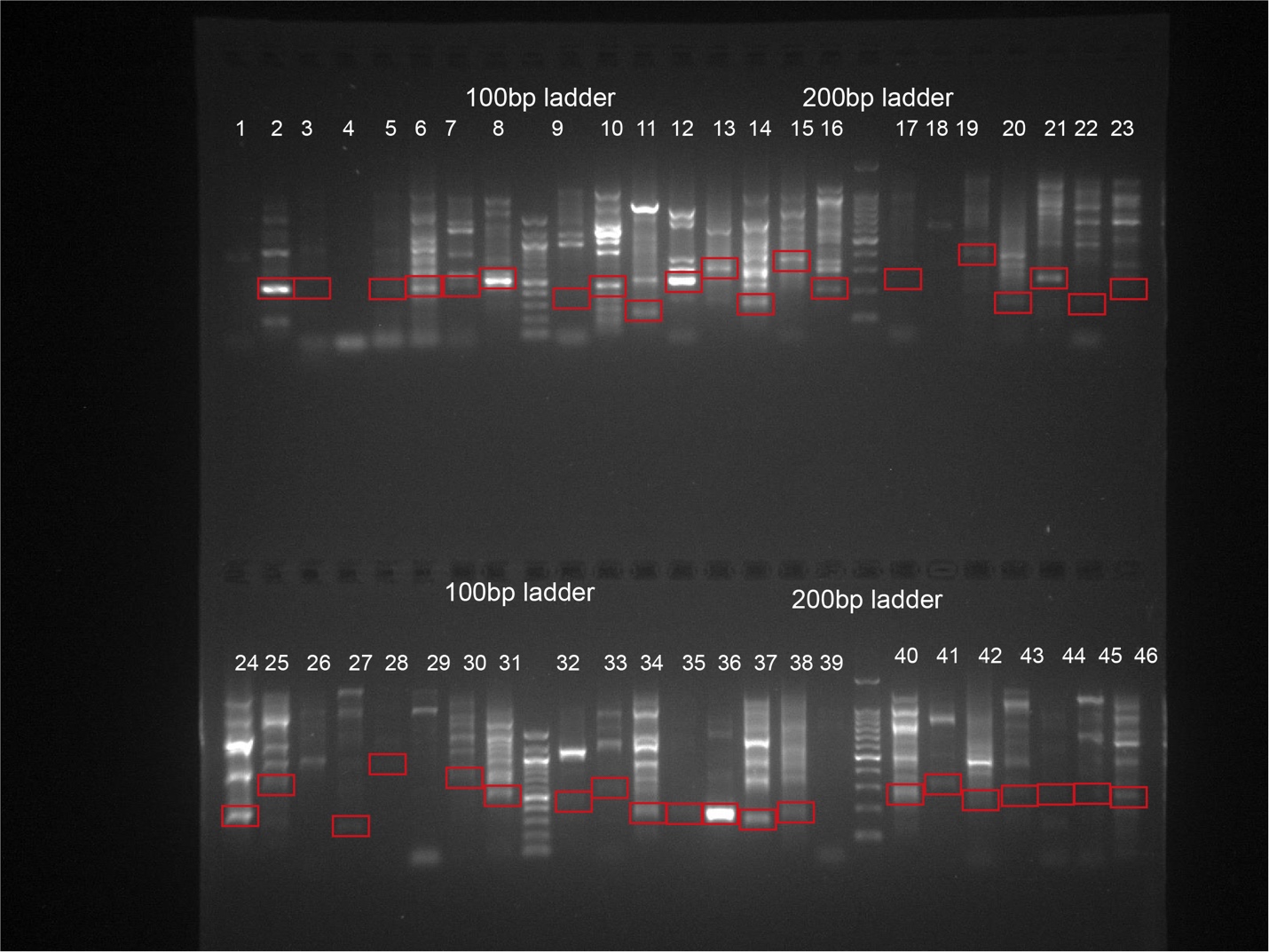


**Figure S2. PCR validation of the identified ecDNAs.** The red box indicates the expected DNA size. We found many other structural variations around the junction region, so the PCR-targeted fragments are not unique.


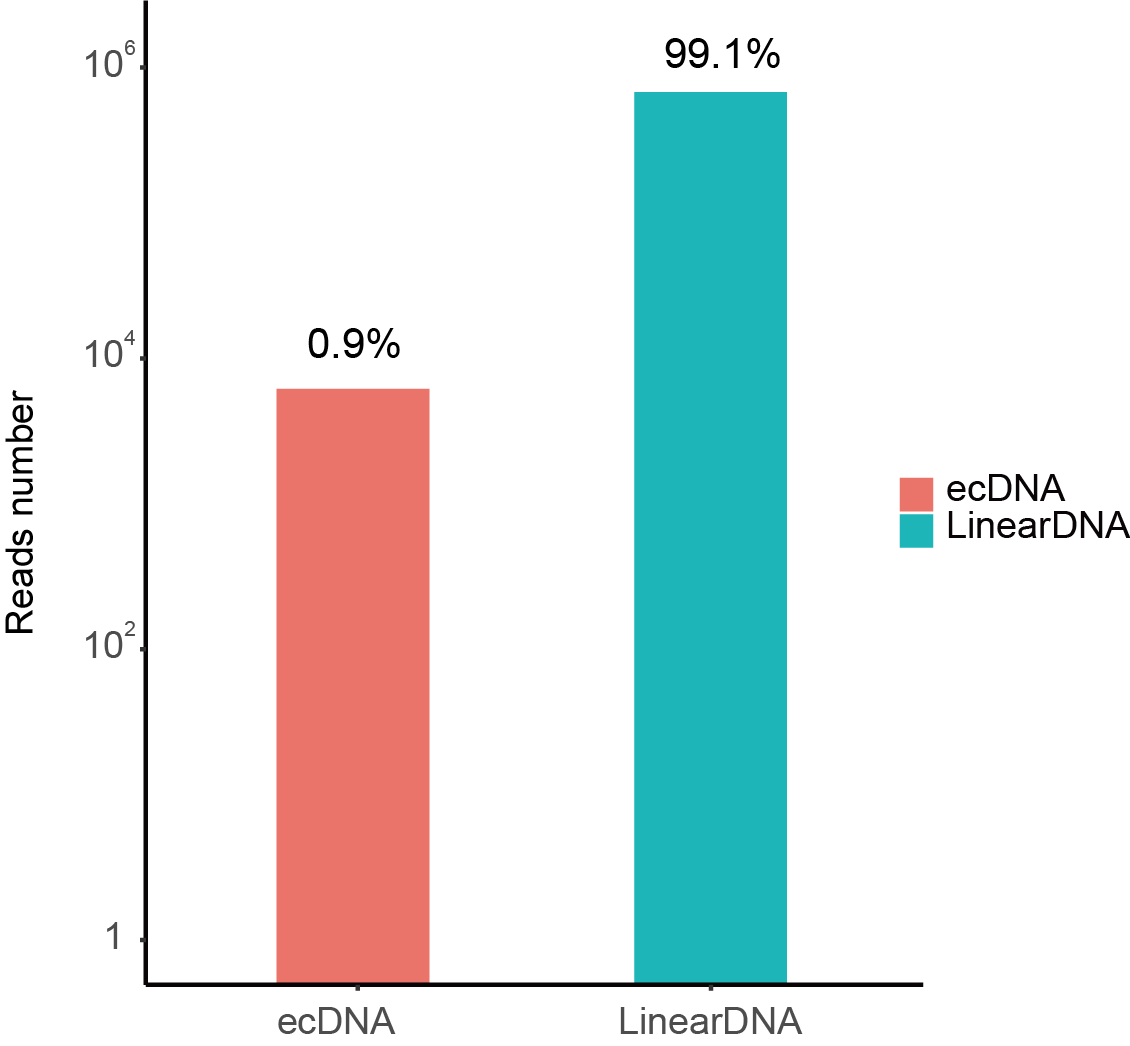


**Figure S3. Counts of reads identified as ecDNA or linear DNA after exonuclease digestion.**


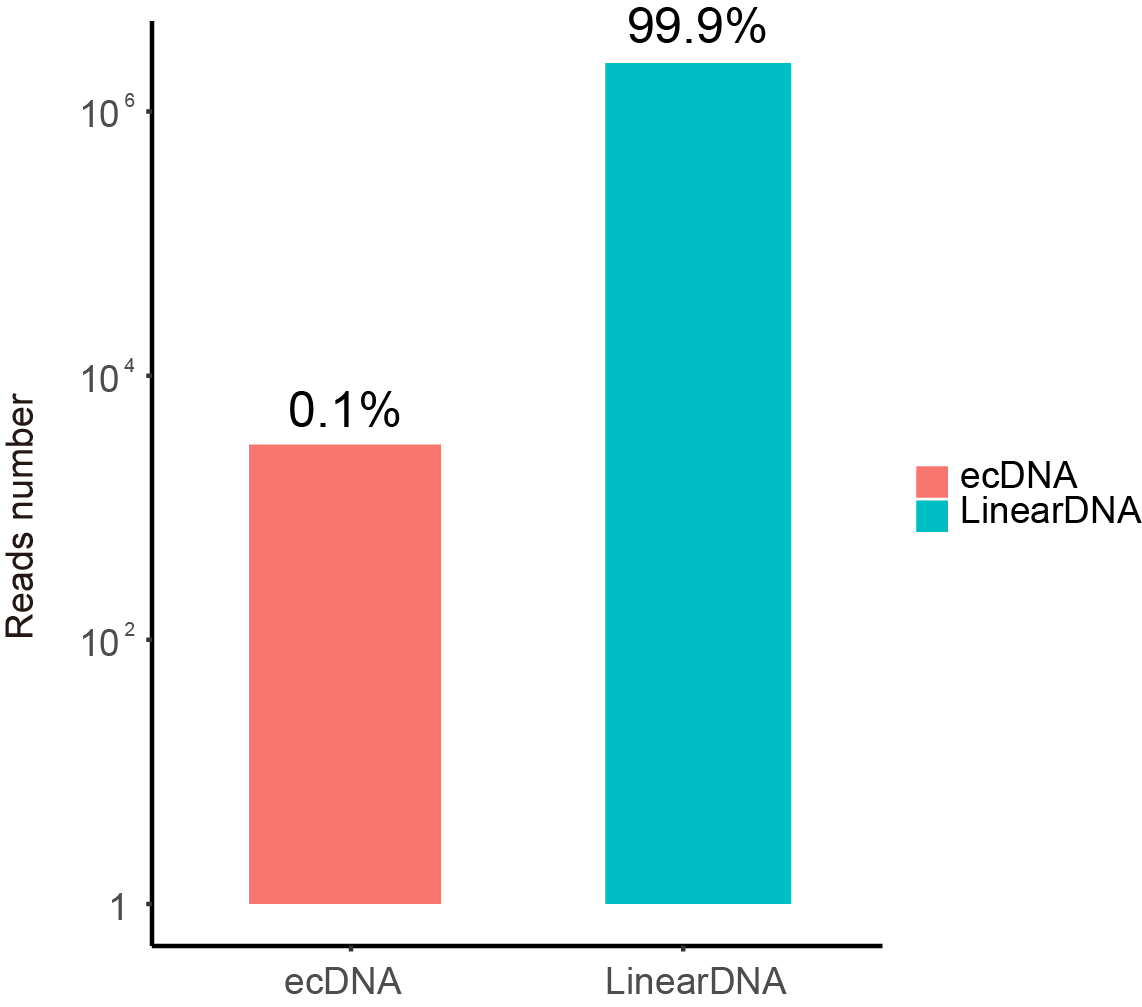


**Figure S4. Counts of reads identified as ecDNA or linear DNA.** The sample was directly subjected to nanopore DNA sequencing without the exonuclease digestion to remove the linear DNA.


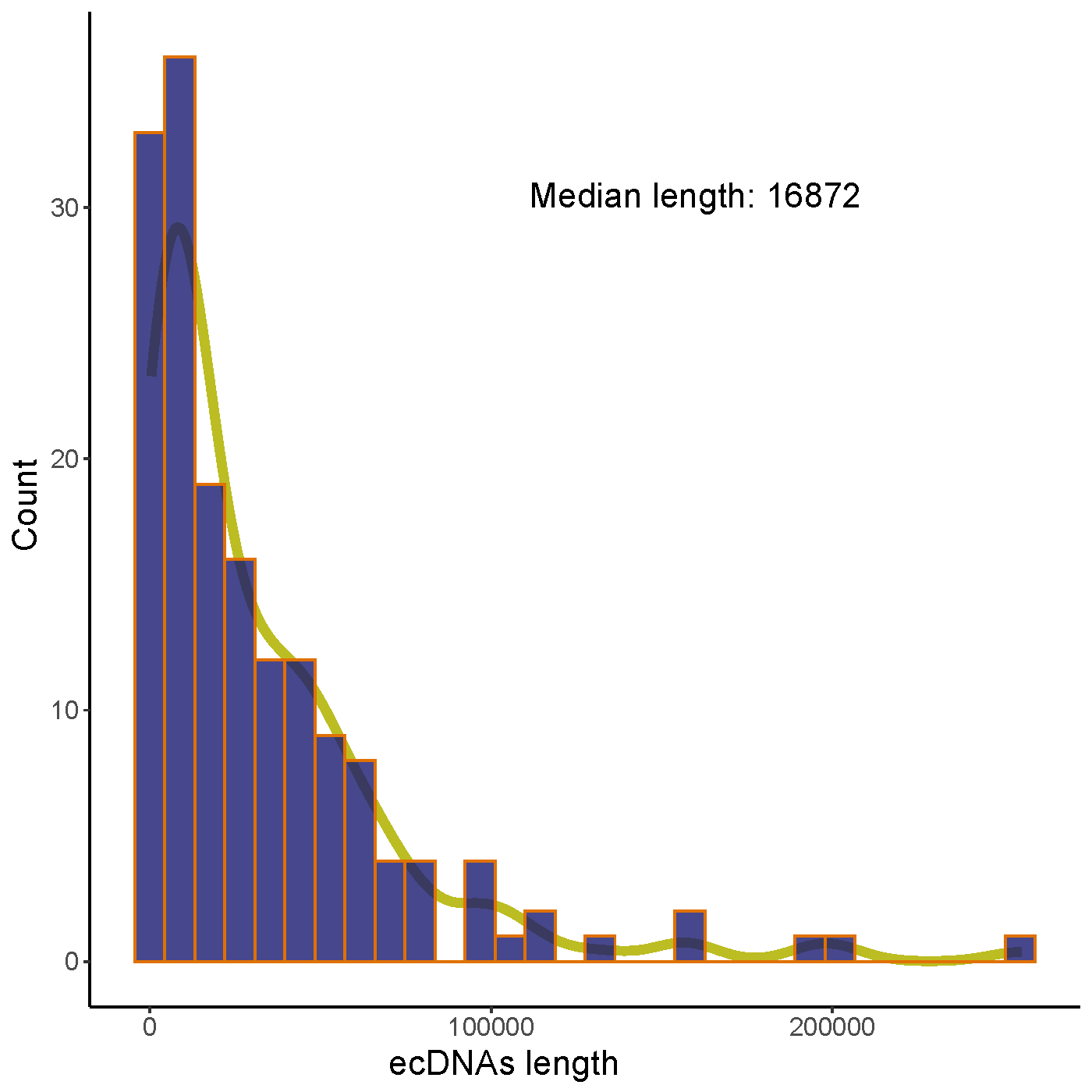


**Figure S5. The lengths of ecDNAs** **assembled with all the covered reads.**

**
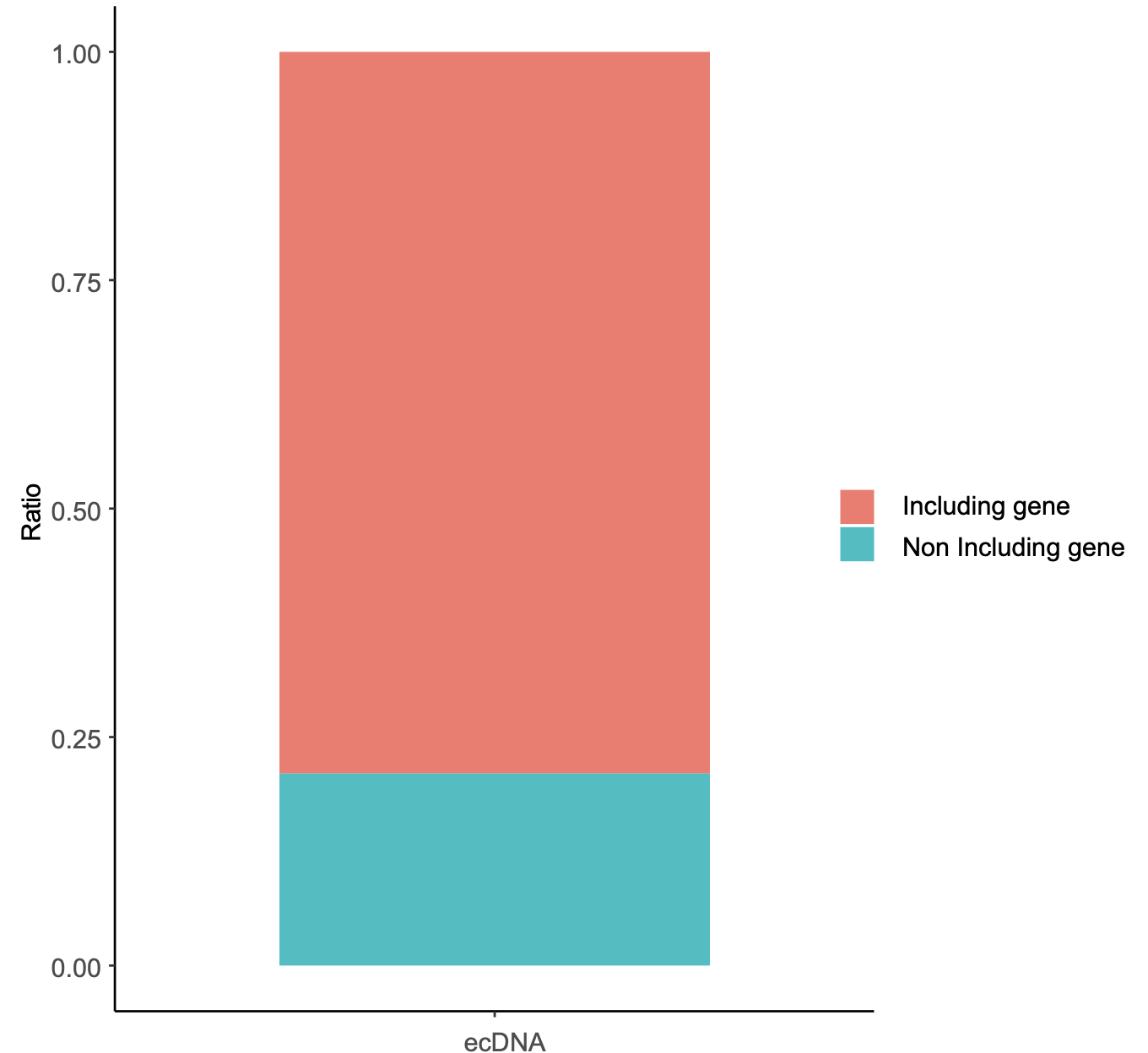
**

**Figure S6. Gene-coding fraction of ecDNAs** **assembled with all covered reads**. Seventy-five percent of reconstructed ecDNAs contained gene coding frames.

**
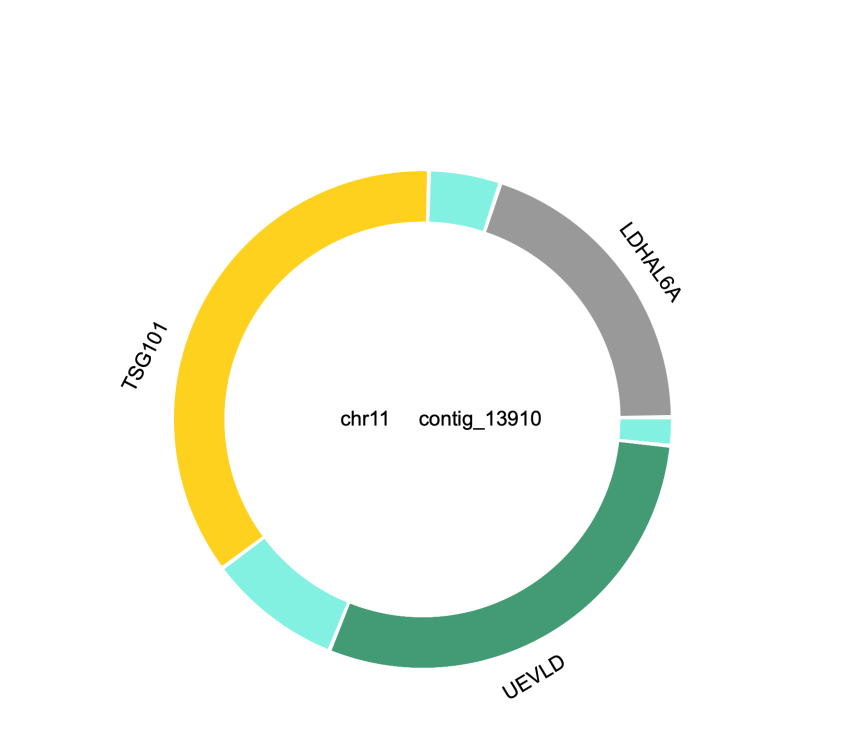
**

**
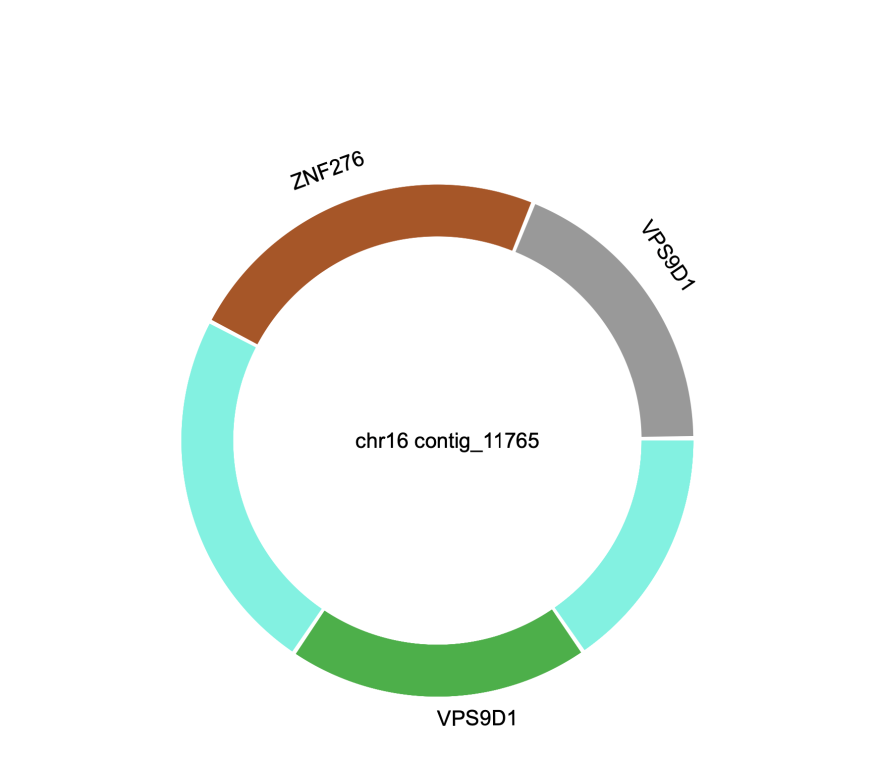
**

**Figure S7. Examples of assembled ecDNAs.** Both ecDNAs contained full oncogene sequences.


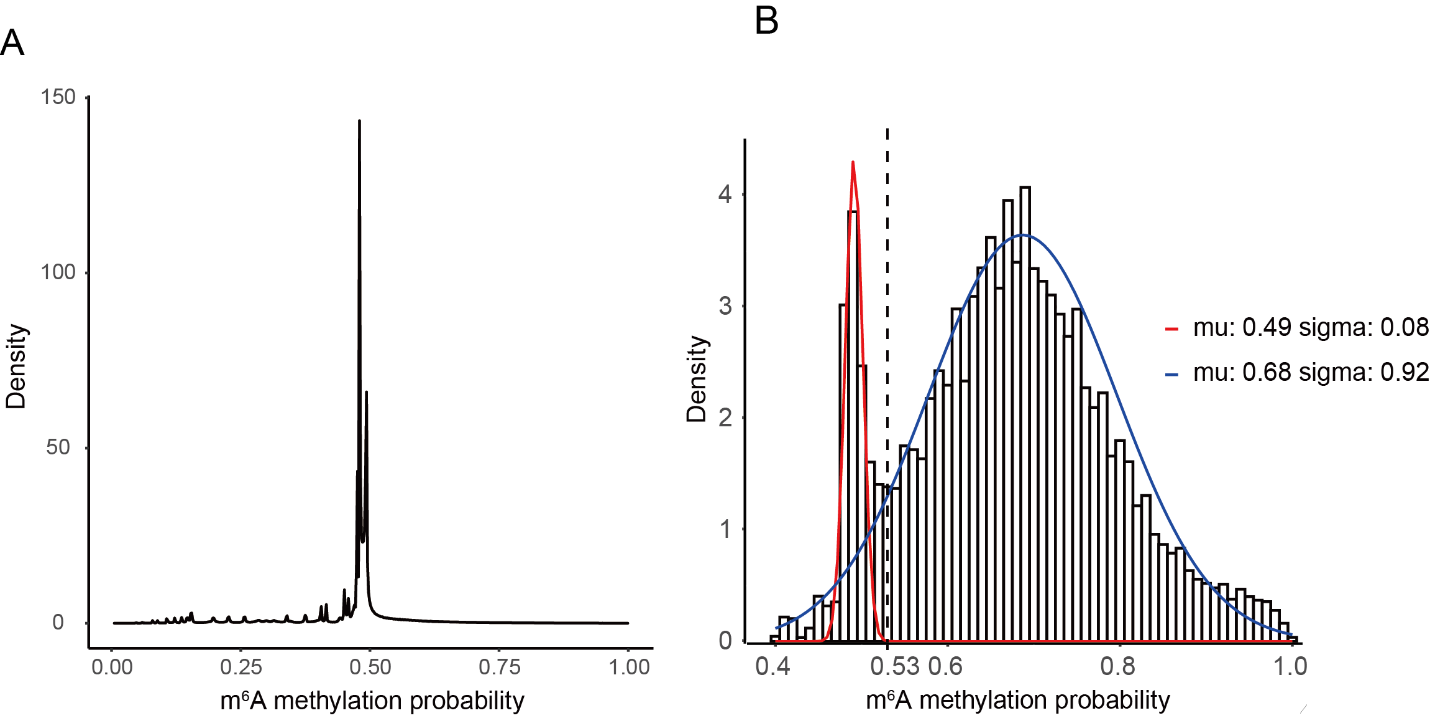


**Figure S8. m6A methylation probability distributions of the treated and non-treated samples.** The signal data were transformed to the m6A sites with possibility of methylation on each site. The m6A probability distribution of the non-treated negative sample, which had no detectable m6A, was below 0.52. The two peaks (0.5 and 0.7) could be classified as positive and negative sites, respectively. The cut-off value was set as 0.53, and the m6A calling specificity and sensitivity were 0.99 and 0.92 respectively.


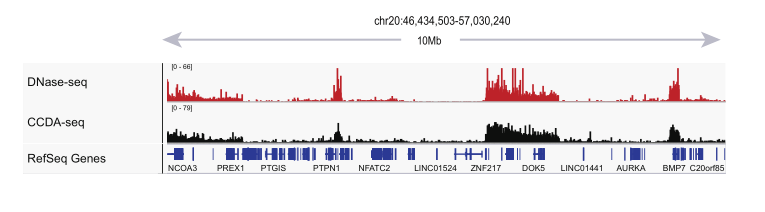


**Figure S9. Large aggregate CCDA-seq signal enrichments match closely DNase-seq accessibility peaks.** (Chr20:46,434,503–57,030,240)


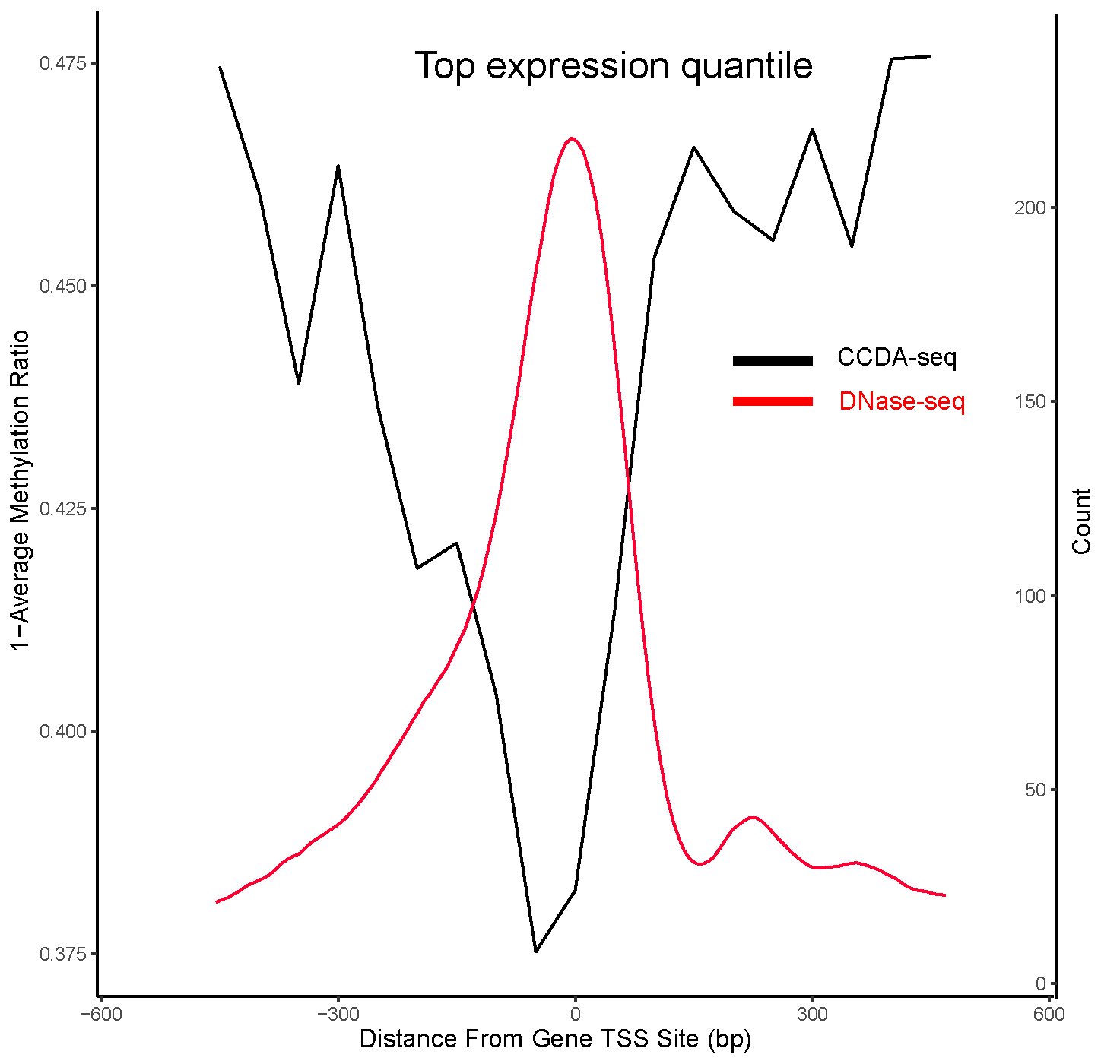


**Figure S10. Chromatin accessibility around the TSSs of the highly expressed genes.** CCDA-seq and DNase-seq demonstrated the similar nucleosome pattern around TSSs of the highly expressed genes.

**
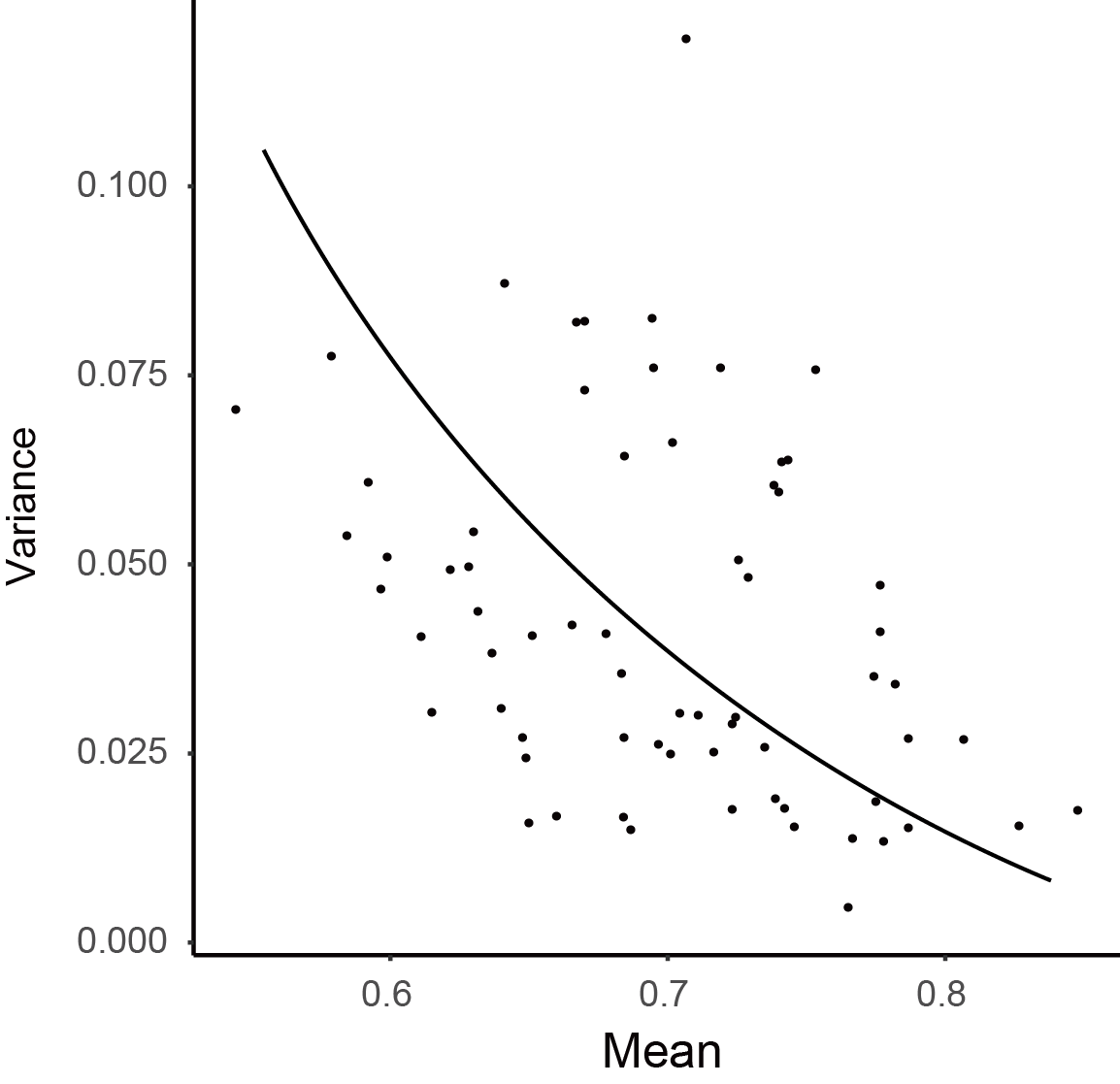
**

**Figure S11. The m6A methylation deviation is related to the average m6A methylation.** The genome was sized into 50 bp bins. The average m6A methylation was calculated as (total m6A in all covered reads)/(total adenosine in all covered reads). The deviation was represented by the bin methylation deviation, which aggregates the methylation in each bin.


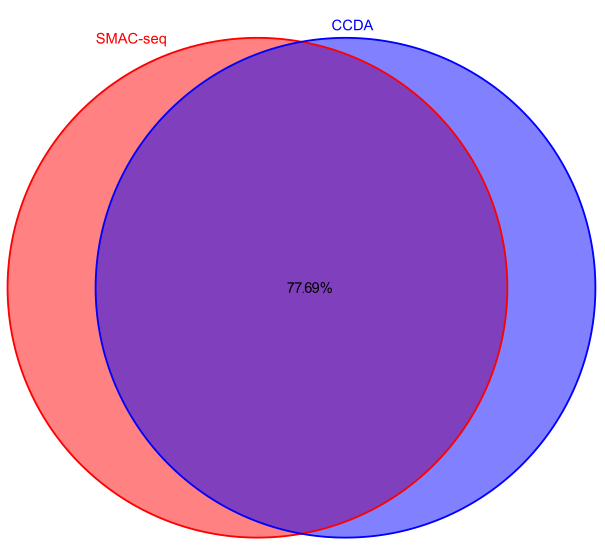


**Figure S12. Correlation between two sample replicates, obtained with and without exonuclease treatment.** The methylated bins (methylated count > 2, bin = 50 bp) overlapped by 77.69% between a non-exonuclease-digested sample that was analyzed by SMAC-seq and an exonuclease-digested sample that was analyzed by CCDA-seq.


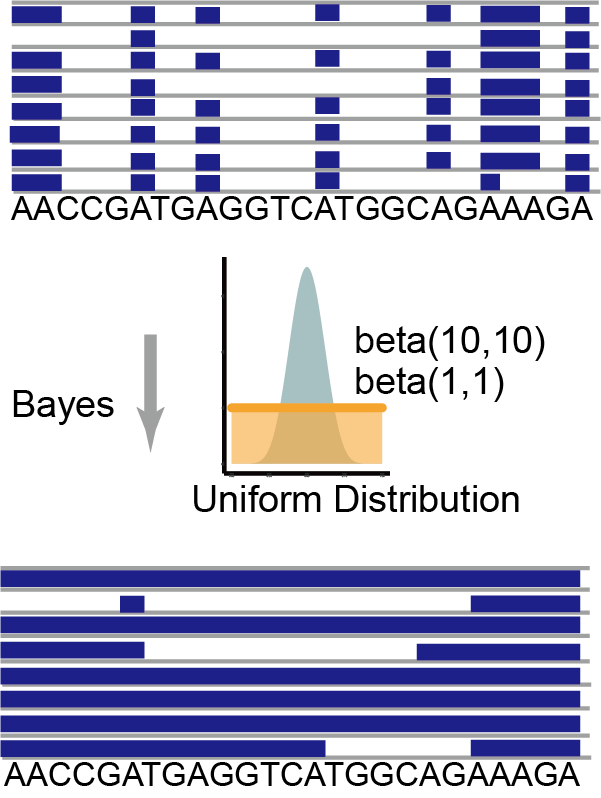


**Figure S13. Outline of the algorithm to configure the methylation level at genome and single-molecule resolutions.** At genome resolution, the methylation fraction represents the average methylation fraction of multiple reads covered in the regions. In the single molecular resolution, we adopted a Bayesian procedure to aggregate methylation probabilities and derived accurate single-molecule accessibility calls over 50 bp windows.


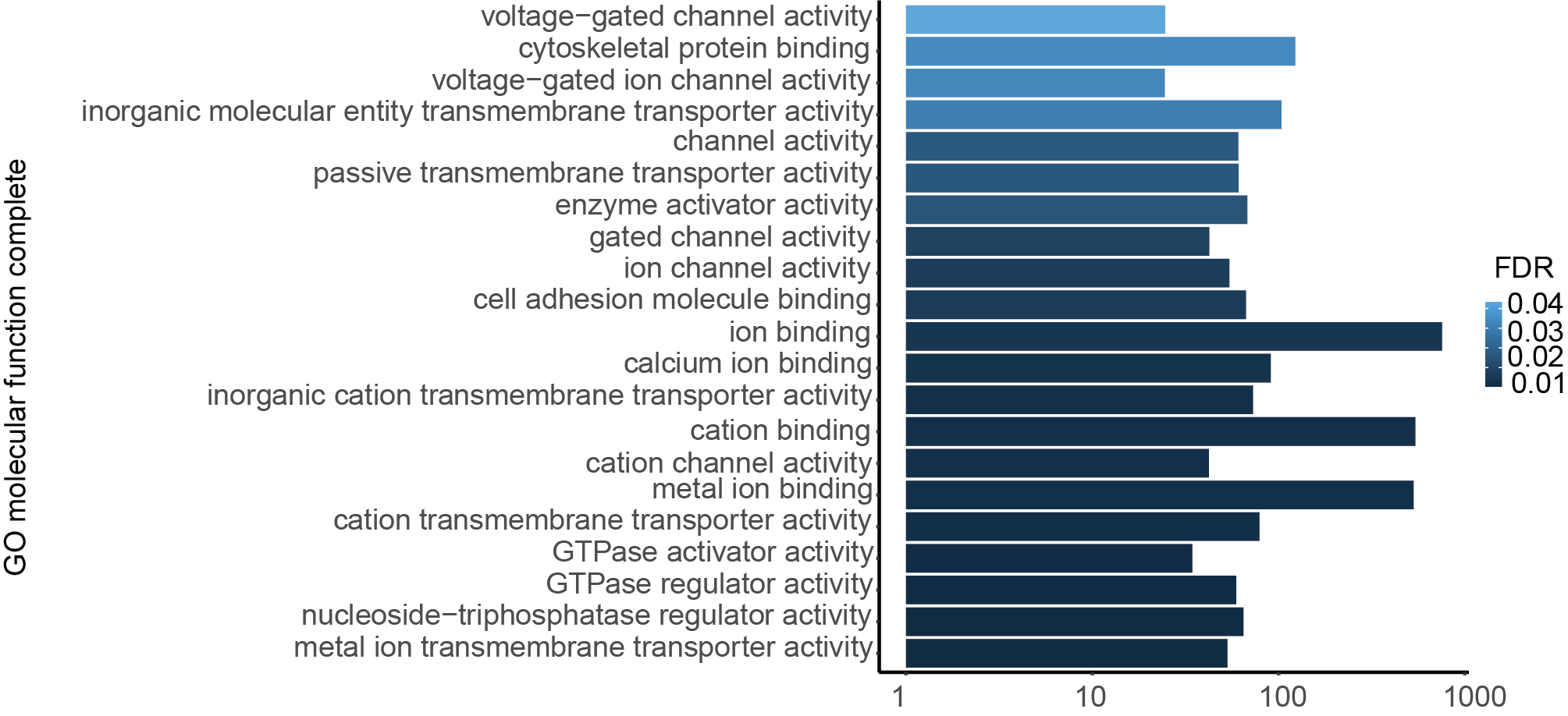


**Figure S14. Gene ontology analysis of the genes encoded by ecDNA** (<http://geneontology.org/>).


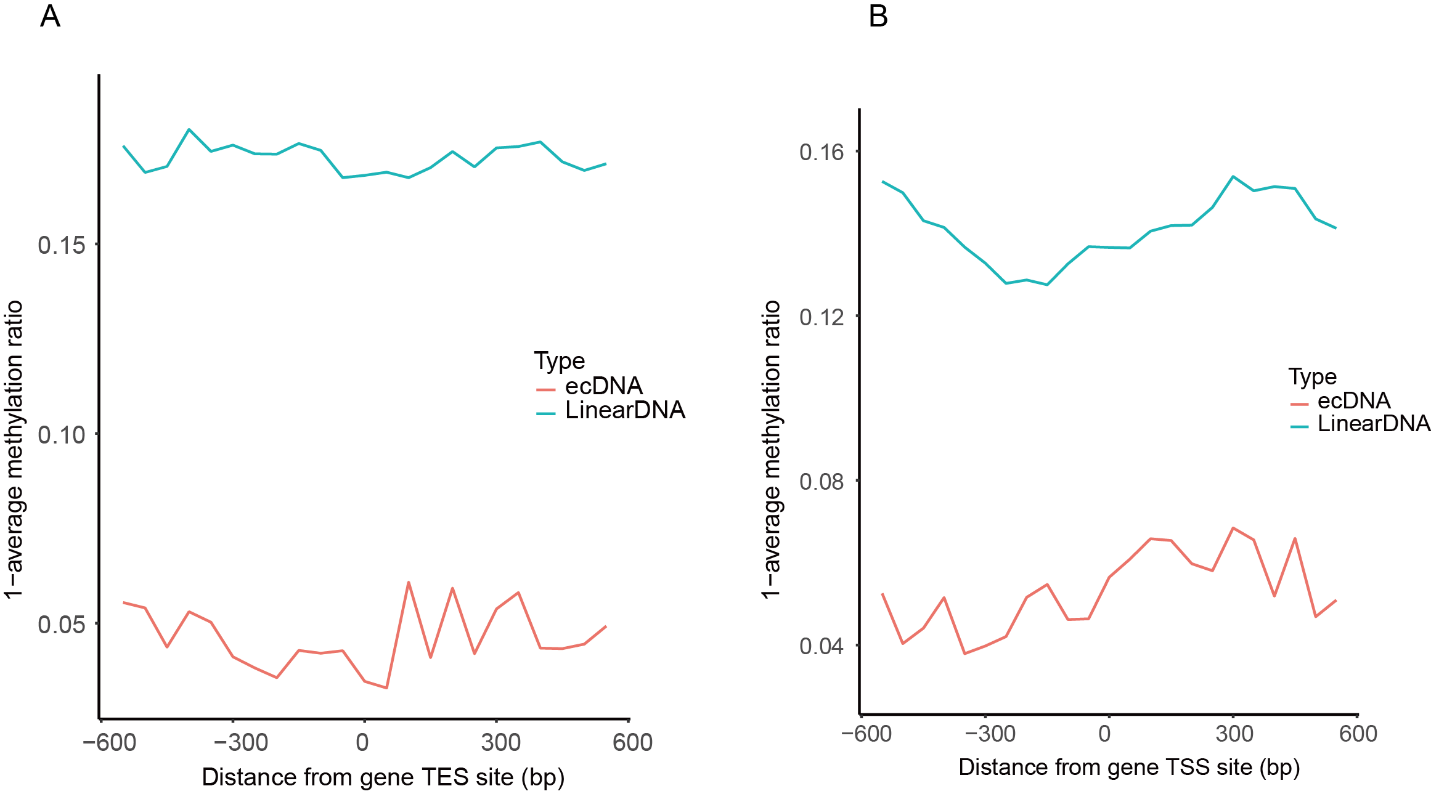


**Figure S15. Average CCDA-seq profiles around TSSs and TES of group II genes.**  ecDNA group II genes had more open chromatin structure the those in the linear DNA (Figure 2C).


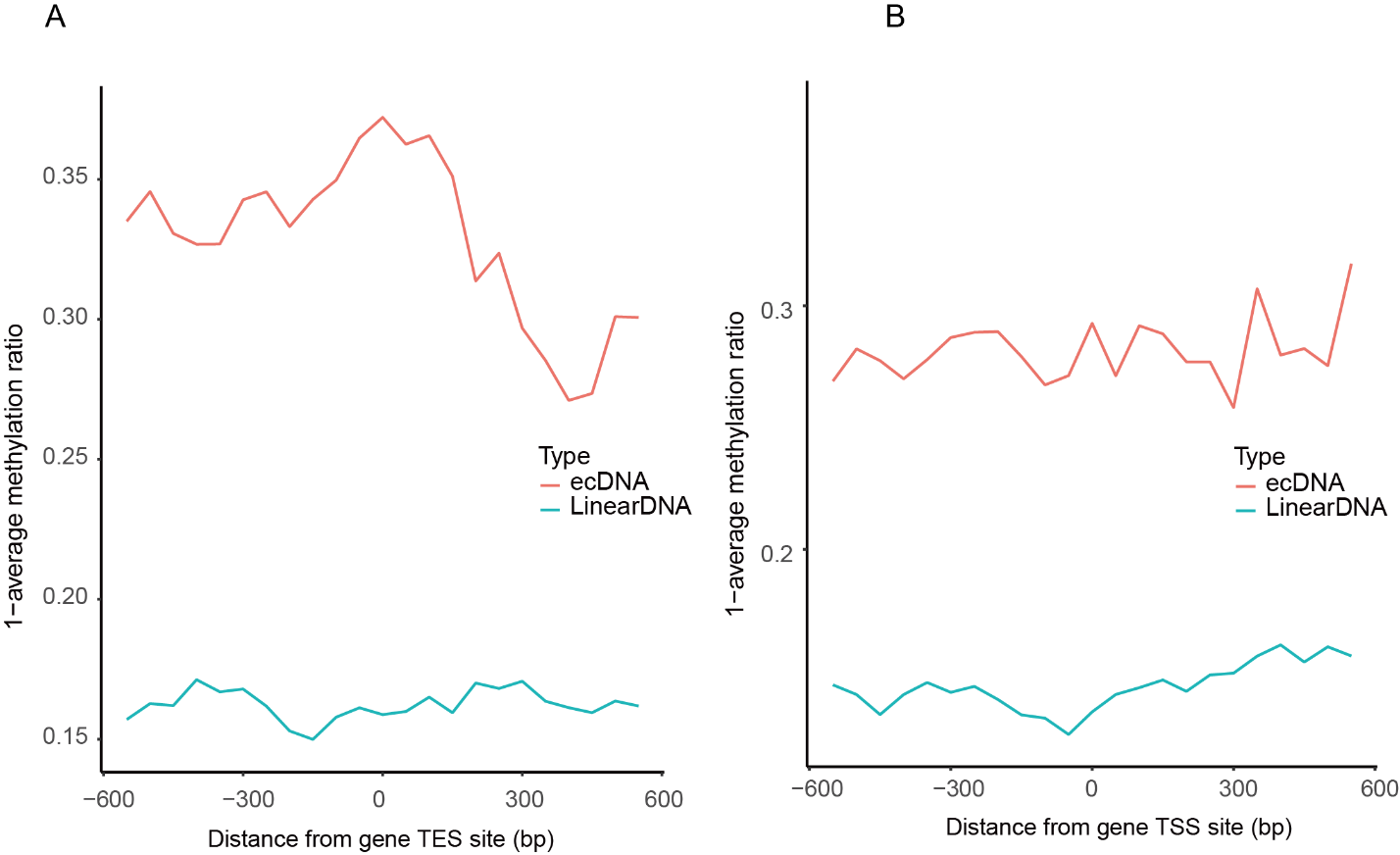


**Figure S16. Average CCDA-seq profiles around TSSs and TES of group I gene.** Linear DNA group I genes have more open chromatin structure than those in the ecDNA (Figure 2C).


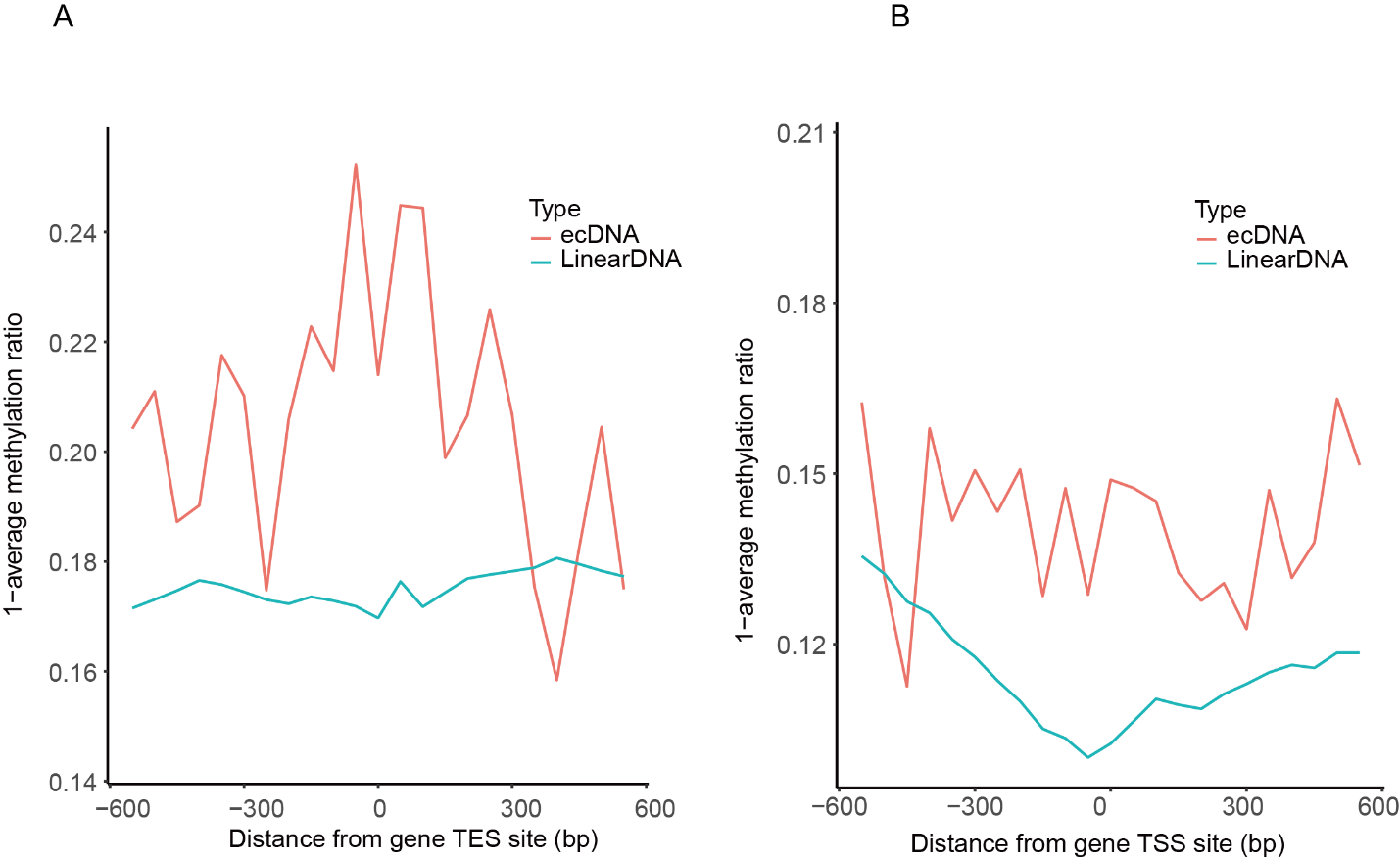


**Figure S17. Average CCDA-seq profiles around TSSs of the highly expressed genes.** The top quantile defines genes with high level of expression in RNA-seq.


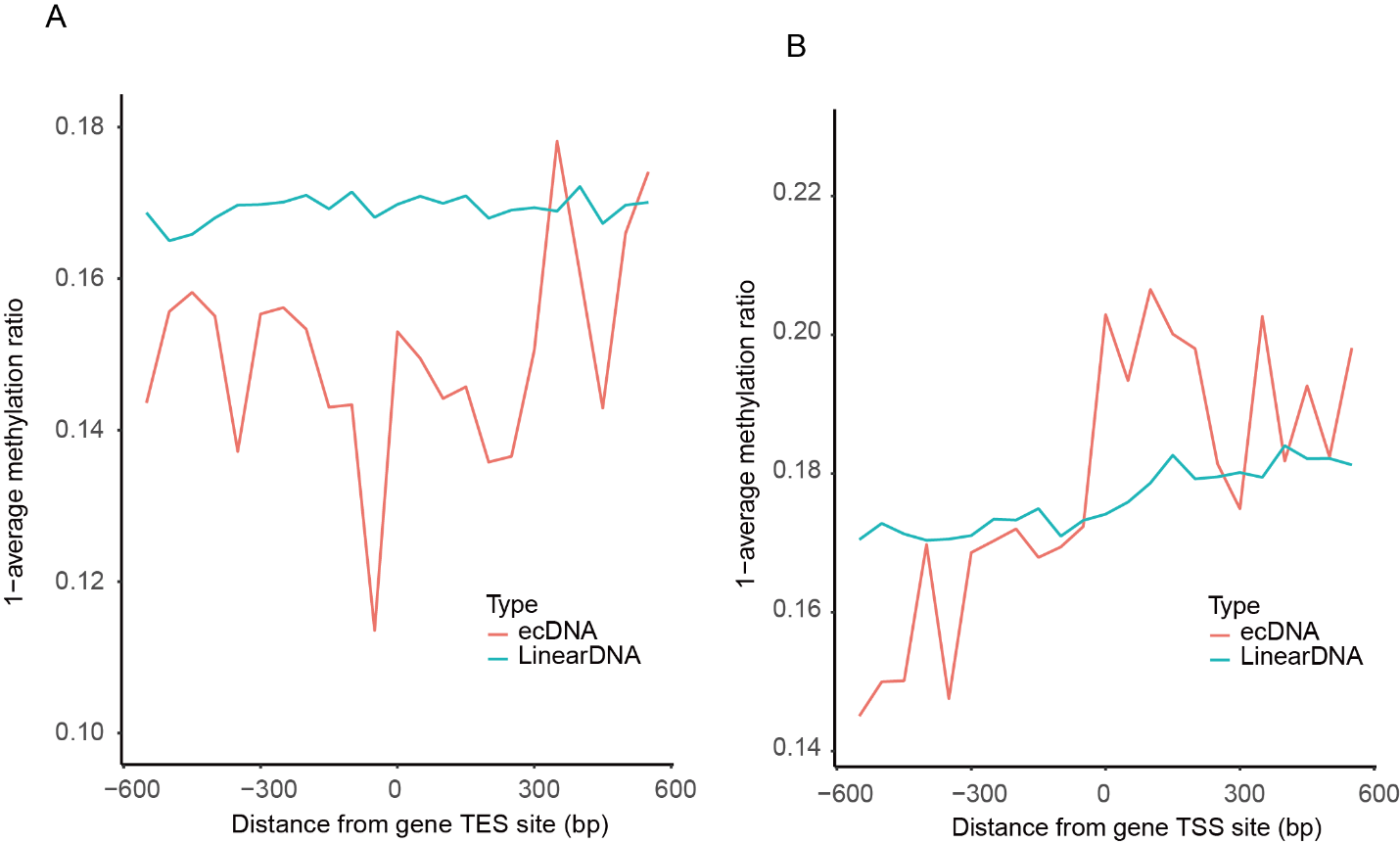


**Figure S18. Average CCDA-seq profiles around TSSs of genes with low expression levels.** The bottom quantile defines genes with low level of expression in RNA-seq.


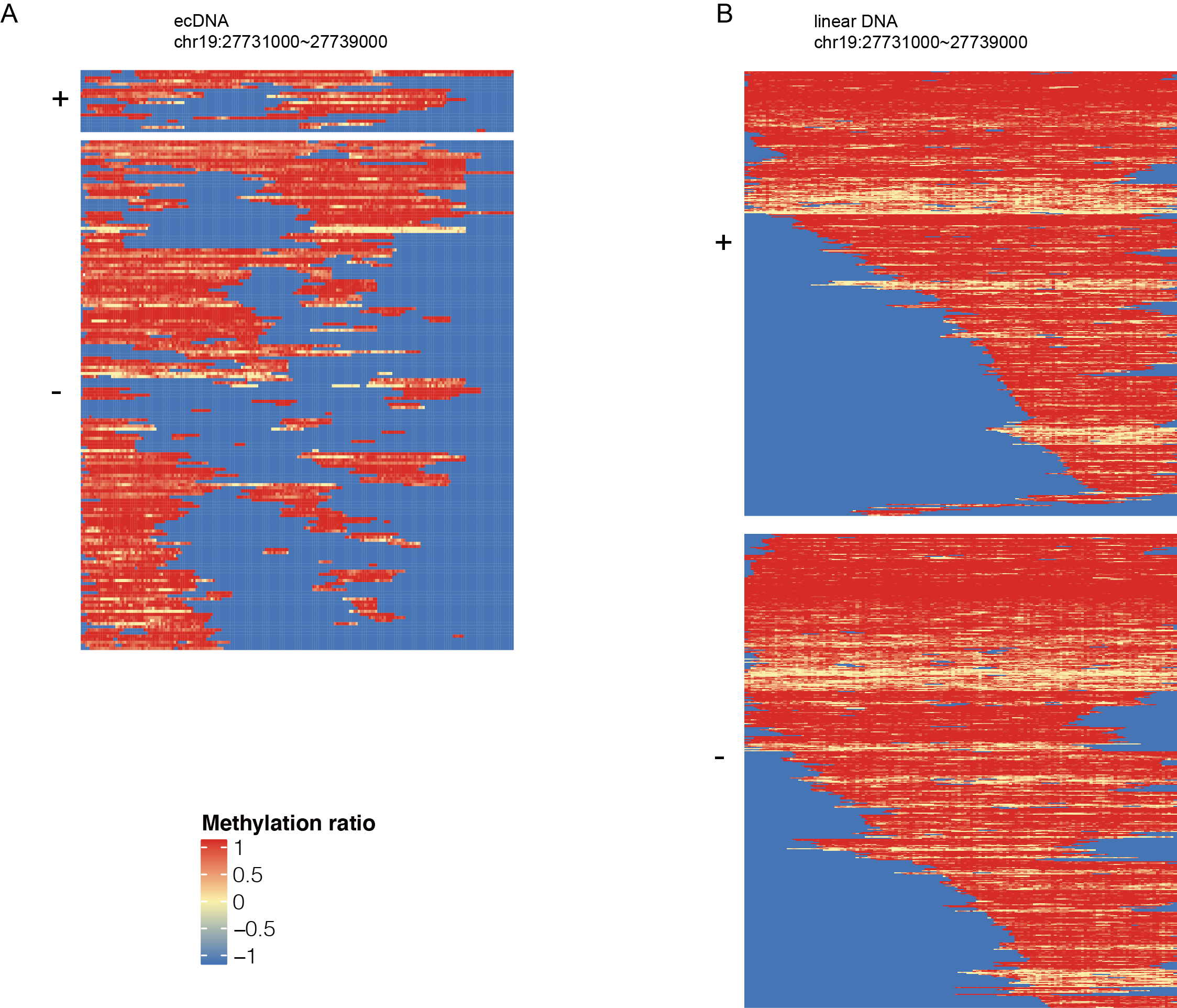


**Figure S19. CCDA-seq reveals the distribution of alternative chromatin states at a single molecule resolution.** A. Shown are all reads (+/-) covering linear DNA regions. B. Shown are all reads (+/-) covering ecDNA regions.


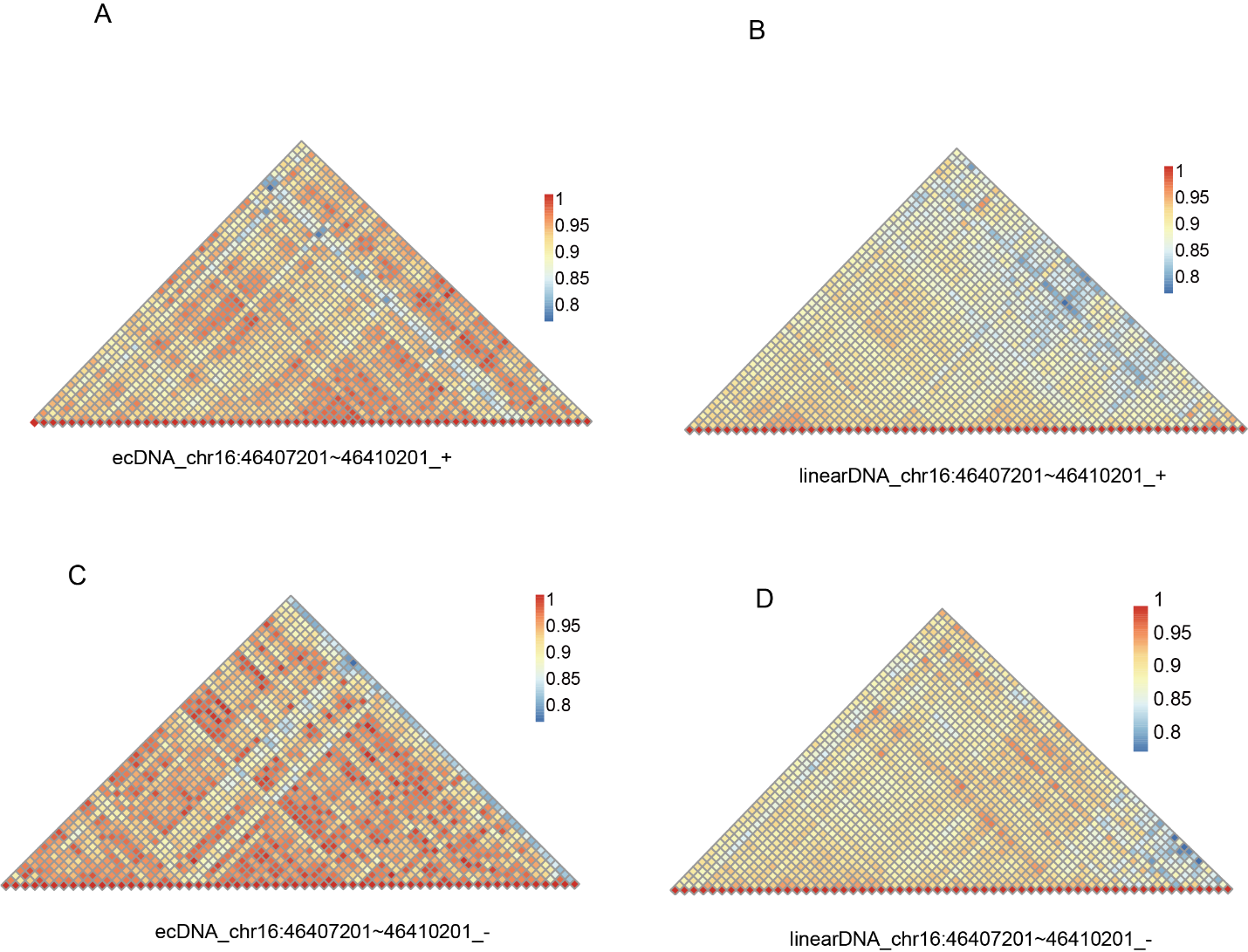


**Figure S20. Chromatin co-accessibility profiles for chr16:46407201–46410201 show correlation and anticorrelation in the ecDNA and linear DNA positive strands** **(A, B) and negative strands (C, D), respectively**.


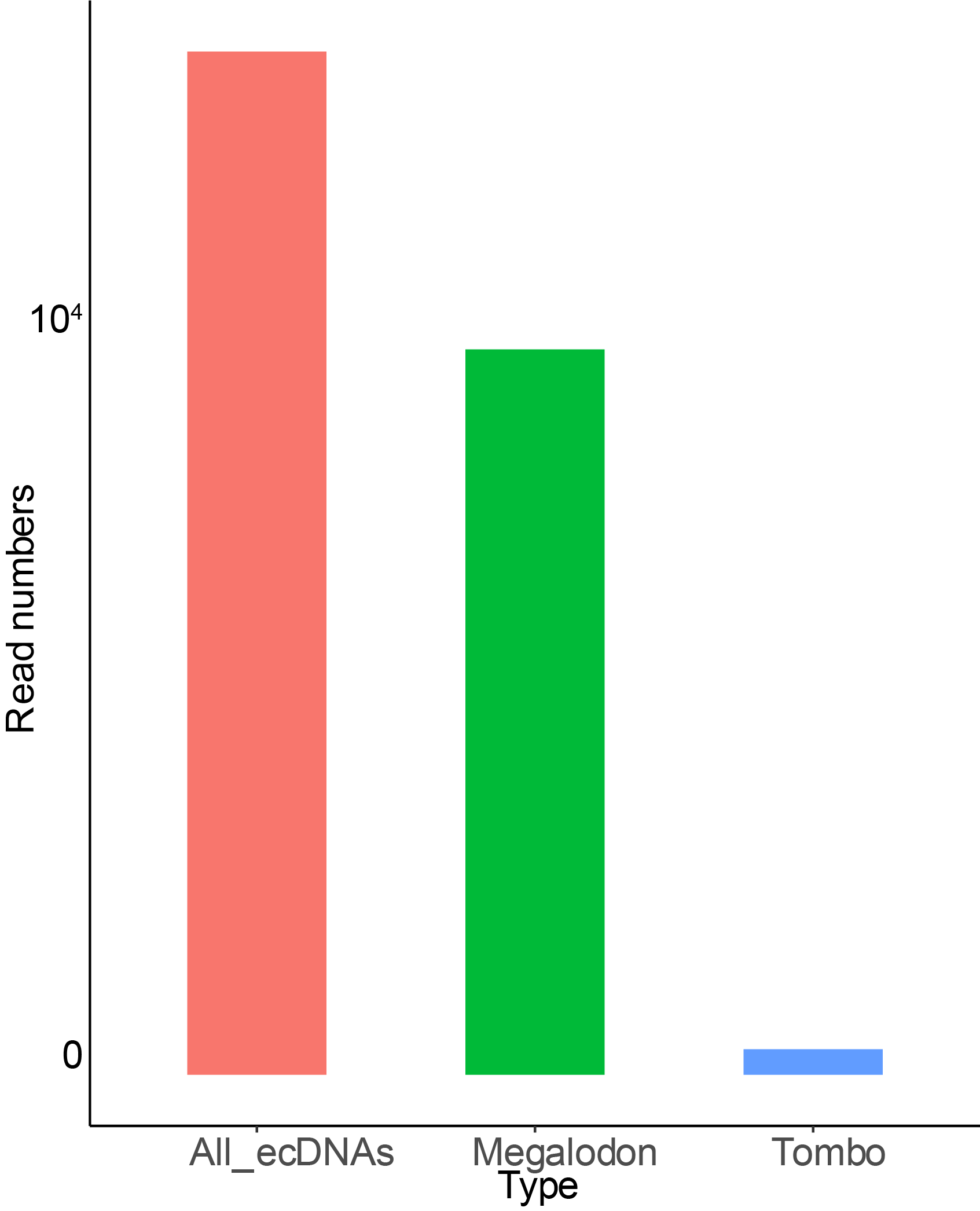


**Figure S21. Comparison of Megalodon and Tombo in ecDNA methylation calling. “**All_ecDNAs” indicates ecDNAs identified by minimap2. All detected ecDNAs were processed for methylation calingl by Megalodon or Tombo.

**
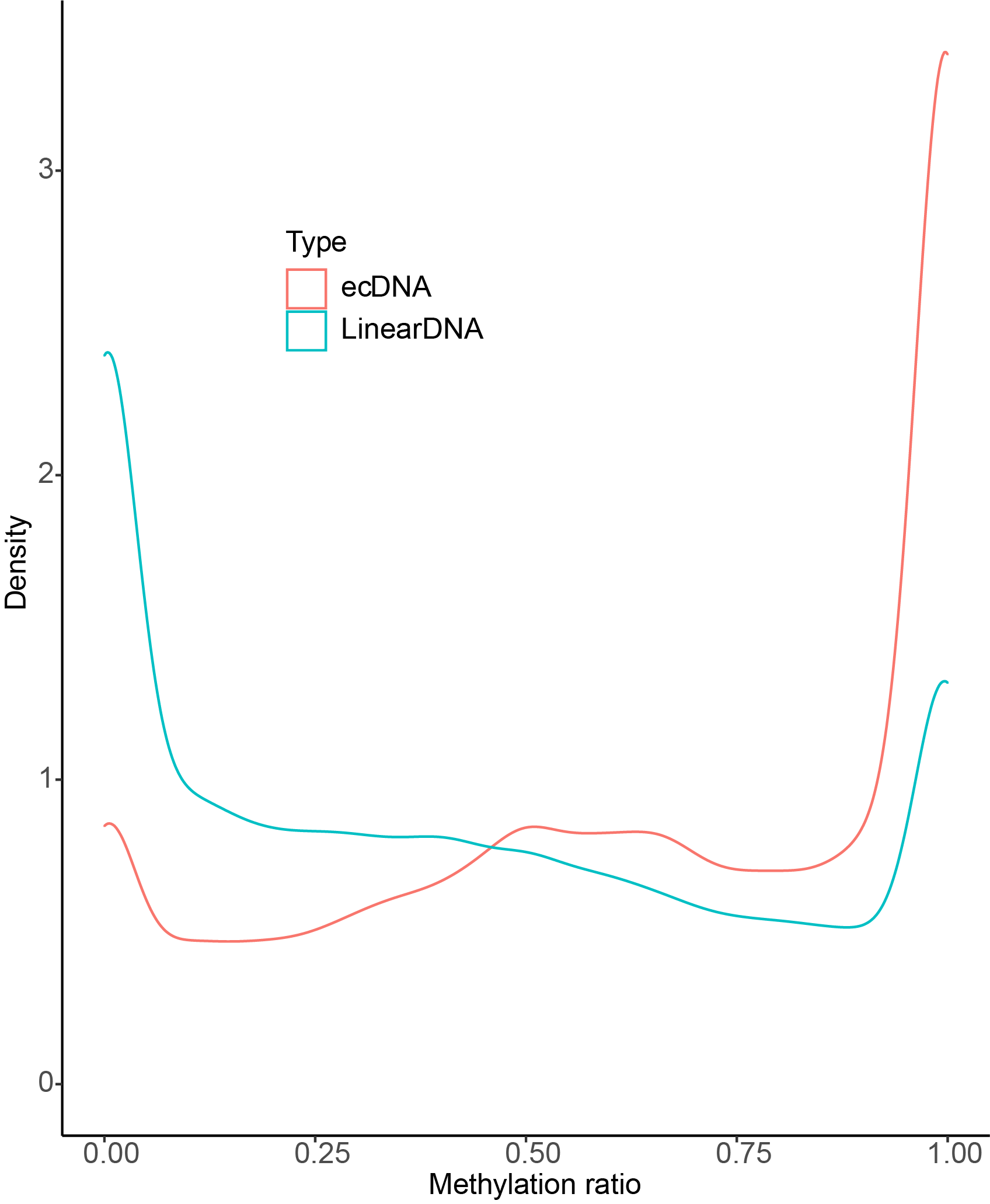
**

**Figure S22. The density distribution of the methylation ratio in ecDNA and linear DNA in the non-exonuclease digested sample**

**
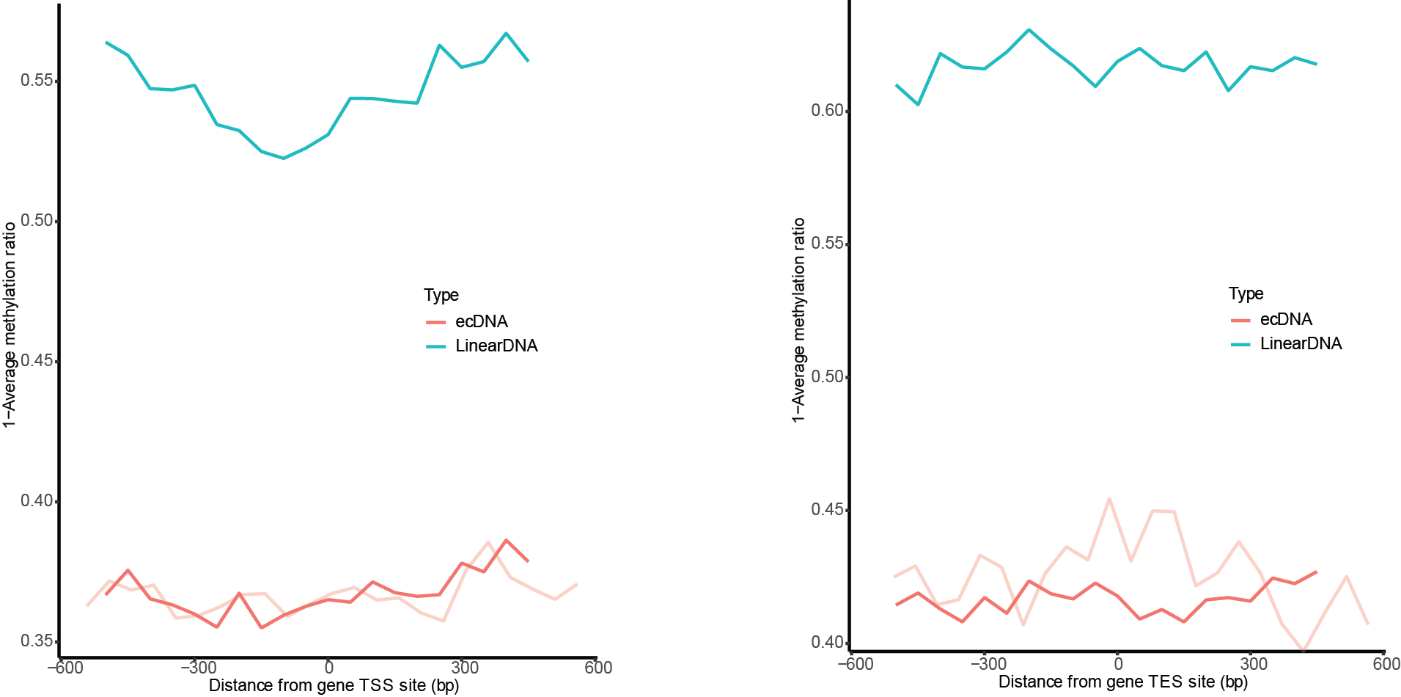
**

**Figure S23. Average CCDA-seq profiles (no linear DNA digestion) around the TSS and TES in ecDNAs and linear DNAs.** The sample, not digested by exonuclease, showed lower average methylation of both ecDNAs and linear DNAs. The light red indicated nucleosome occupancy in the CCDA-seq after exonuclease digestion. Approximately similar trends were noted between the non-digested sample and digested sample. The difference may be caused by the distinct ecDNA coverage in the two samples. The exonuclease treatment did not bias our analysis (aggregated over 50-bp windows sliding every 5 bp; the sequencing depth was normalized for the ecDNA and linear DNA; see Methods for details)

**
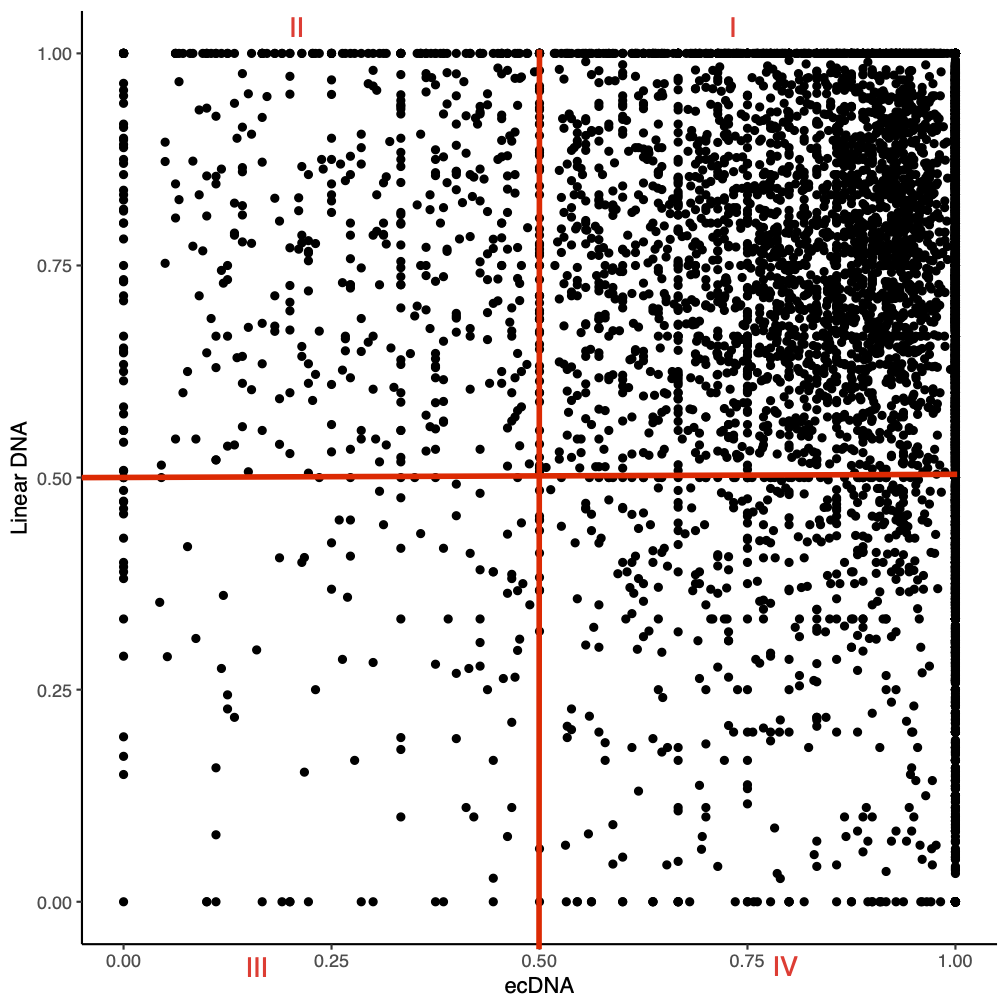
**

**Figure S24. Pairwise scatter plot of the average bin methylation in the ecDNA and linear DNA.** The genome is sized into 50 bp bins. The methylation in each bin is the methylation ratio average of covering reads. The bins were classified into four groups: group I - highly accessible regions in both linear DNA and ecDNAs; group II - linear DNA regions less accessible than ecDNA regions; group III - regions inaccessible in both linear DNA and ecDNA; group IV - ecDNA regions with more open chromatin than that in the linear DNA areas.


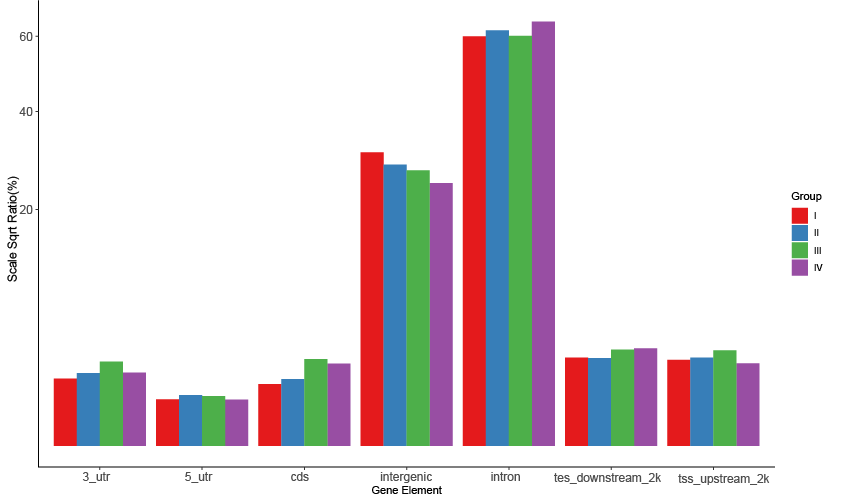


**Figure S25. The bins distribution among gene elements.** The four groups of regions are defined in Supplemental Figure 18.
